# Restoration of female fertility in *Trichoderma reesei* QM6a provides the basis for inbreeding in this industrial cellulase producing fungus

**DOI:** 10.1186/s13068-015-0311-2

**Published:** 2015-09-24

**Authors:** Rita Linke, Gerhard G. Thallinger, Thomas Haarmann, Jasmin Eidner, Martina Schreiter, Patrick Lorenz, Bernhard Seiboth, Christian P. Kubicek

**Affiliations:** ACIB GmbH, c/o Institute of Chemical Engineering, Technische Universität Wien, Gumpendorferstraße 1a, 1060 Vienna, Austria; Bioinformatics, Institute for Knowledge Discovery, Graz University of Technology, Petersgasse 14/V, 8010 Graz, Austria; AB Enzymes GmbH, Feldbergstrasse 78, 64293 Darmstadt, Germany; Research Division Biotechnology and Microbiology, Institute of Chemical Engineering, Technische Universität Wien, Gumpendorferstraße 1a, 1060 Vienna, Austria; Core Facility Bioinformatics, ACIB GmbH, Petersgasse 14/V, 8010 Graz, Austria

**Keywords:** *Trichoderma reesei*, Female fertility, Cellulase, Comparative genomics, Strain breeding

## Abstract

**Background:**

Filamentous fungi are frequently used as production platforms in industrial biotechnology. Most of the strains involved were known as reproducing exclusively asexually thereby preventing the application of conventional strain breeding techniques. In the last decade, evidence was obtained that a number of these imperfect fungi possess a sexual life cycle, too. *Trichoderma reesei,* an industrial producer of enzymes for food, feed and biorefinery purposes, is heterothallic and takes a special position among industrially utilized species as all industrial strains are derived from the single *MAT1*-*2* isolate QM6a. Consequently, strain improvement by crossing is not feasible within this strain line as this necessitates a *MAT1*-*1* mating partner. Simply switching the mating type in one of the mating partners to *MAT1*-*1,* however, is not sufficient to produce a genotype capable of sexual reproduction with QM6a *MAT1*-*2*.

**Results:**

We have used a systems biology approach to identify genes restoring sexual reproduction in the QM6a strain line. To this end, *T. reesei* QM6a was crossed with the *MAT1*-*1* wild-type strain CBS999.97. The descendants were backcrossed 8-times in two lineages with QM6a to obtain mating competent *MAT1*-*1* strains with a minimal set of CBS999.97 specific genes. Comparative genome analysis identified a total of 73 genes of which two—encoding an unknown C2H2/ankyrin protein and a homolog of the WD-protein HAM5—were identified to be essential for fruiting body formation. The introduction of a functional *ham5* allele in a mating type switched *T. reesei* QM6a allowed sexual crossing with the parental strain QM6a.

**Conclusion:**

The finding that *Trichoderma reesei* is generally capable of undergoing sexual reproduction even under laboratory conditions raised hope for the applicability of classical breeding techniques with this fungus as known for plants and certain yeasts. The discovery that the wild-type isolate QM6a was female sterile, however, precluded any progress along that line. With the discovery of the genetic cause of female sterility and the creation of an engineered fertile strain we now provide the basis to establish sexual crossing in this fungus and herald a new era of strain improvement in *T. reesei*.

**Electronic supplementary material:**

The online version of this article (doi:10.1186/s13068-015-0311-2) contains supplementary material, which is available to authorized users.

## Background

Filamentous fungi are key organisms in biotechnology, being at the core of many industrial processes, such as the production of enzymes, vitamins, polysaccharides, polyhydric alcohols, pigments, lipids, organic acids and glycolipids [1, 2]. Strain improvement of these organisms to obtain commercially acceptable production yields has for a long time been mostly relying on classical mutagenesis and only in the last two decades recombinant DNA technologies increasingly gained importance. This is because—in contrast to plants and yeasts, for which strain breeding by crossing has been an essential strategy for strain improvement—the majority of them were isolated in their asexual form and their sexual form and life cycle had been unknown for many years. In the last decade, evidence was obtained that also a number of these fungi possess a sexual cycle [3–5]. The ability to mate industrially relevant fungi under laboratory conditions has been demonstrated, amongst others, for the organic acid producer *Aspergillus tubingensis* [6] and the penicillin producer *Penicillium chrysogenum* [7]. Crossing of different industrial strains of a given species would have the significant advantage of being a “natural” strategy for rapidly introducing multiple favorable traits even if their genetic foundation is unknown or to remove mutations or undesired genes like secondary metabolite clusters or antibiotic resistance markers whose presence may interfere with regulatory requirements.

*Trichoderma reesei* (*syn. Hypocrea jecorina*) is a major industrial enzyme producer, particularly of cellulases and hemicellulases that are used for applications in the food, feed and biorefinery businesses [8–11]. This fungus too was for a long time considered to be asexual. Seidl et al. [12] first described the ability of *T. reesei* to perform sexual reproduction, and to be heterothallic. Heterothallic species require two compatible mating partners each of them carrying a different mating type locus—either *MAT1*-*1* or *MAT1*-*2*—which are required for successful sexual reproduction [13, 14].

All strains of *T. reesei* which are nowadays used in industry and most strains used in academic research originate from a single isolate—*T. reesei* QM6a [15] which carries the mating type *MAT1*-*2.* This implies that for crossing industrial lineages of these enzyme producers, a mating type switch from *MAT1*-*2* to *MAT1*-*1* is needed in one of the mating partners. However, a simple mating type switch proved to be insufficient to enable sexual reproduction within the *T. reesei* QM6a strain line [12]. This finding is in contrast to, e.g., *Magnaporthe grisea* or *Neurospora crassa*, where strains in which the *MAT* locus was exchanged were fertile in crossings with the respective parental strains [16, 17]. Interestingly, the mating type switched *T. reesei* QM6a strain was able to mate with *MAT1*-*2* wild isolates other than QM6a. From these findings Seidl et al. [12] concluded that *T. reesei* QM6a is very well able to act as a male partner in crossings but that it cannot produce fruiting bodies and hence is female sterile, probably because of its maintenance in laboratories for over 60 years without selective pressure to sustain mating competence.

In this study, we describe the identification of the cause for the female sterility of *T. reesei* QM6a. By comparing the genomes of female fertile *MAT1*-*1* QM6a offspring—obtained after repeated backcrossing with QM6a—to that of their parental strain QM6a, we identified genes putatively required for female fertility and being either mutated or lacking in QM6a. Systematic gene knock-out of the candidate genes for female fertility and their reintroduction led to the deciphering of this malfunction on a genetic basis and enables us to rescue female fertility in *T. reesei* QM6a. This puts us now in the position to establish strain breeding programs by sexual crossing of QM6a descendants.

## Results

### Preparation of two *T. reesei MAT1-1* inbred strain lines

In a first attempt to identify genes that are absent or mutated in *T. reesei* QM6a but functional in sexually reproducing wild-type isolates, and thus are potentially responsible for the observed female sterility of strain QM6a, we sequenced and analyzed the genomes of the *T. reesei* strains C.P.K. 1282 (*MAT1*-*1),* C.P.K. 170 (*MAT1*-*1)* and C.P.K. 938 (*MAT1*-*2*). The two *MAT1*-*1* strains were chosen as they reproduce sexually with QM6a and C.P.K. 938 mates with the two *MAT1*-*1* strains. Comparative genome analysis of these strains revealed cumulative sequence differences between them and *T. reesei* QM6a of a magnitude ranging from 21,000 to 141,000 SNVs (single nucleotide variants) in all exon regions preventing the straightforward identification of genes responsible for female sterility. Therefore, we used an inbreeding strategy first to narrow down the number of candidate genes. We crossed QM6a with different available *MAT1*-*1* wild-type strains followed by backcrossing of the *MAT1*-*1* progenies with QM6a to establish inbred strain lines (see Additional file 1: Table S1). Thereby, strain CBS999.97 was identified as the ideal candidate since these crossings were highly reproducible. Fruiting bodies were formed within 5–10 days vs. 10–20 days for the other tested wild-type strains. Furthermore, the sexual fertility of the progenies was highest from crosses with CBS999.97 whereas the progenies of the other crosses lost their sexual fertility to cross with QM6a after one or two generations.

*MAT1*-*1* progenies from the cross of QM6a and CBS999.97 were backcrossed over eight generations in two separate lines with QM6a, resulting in the two *MAT1*-*1* inbred strains RL1/A8-02 and RL2/A8-11 that were phenotypically similar to QM6a. These two strains were still able to mate with different *MAT1*-*2* wild-type strains (see Additional file 2: Figure S1) as well as with strain QM6a.

### Genome analysis of *T. reesei* RL1/A8-02 and RL2/A8-11

**Fig. 1:**
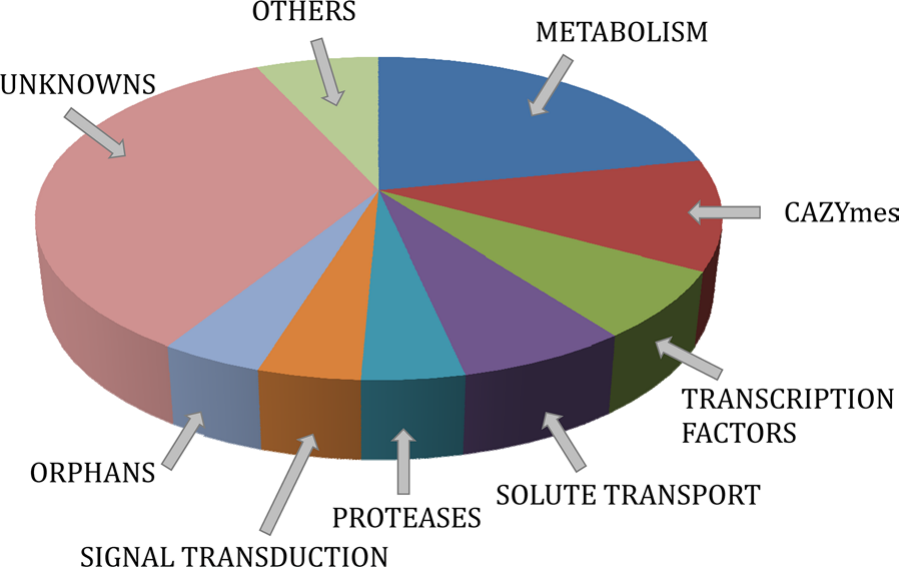
FunCat categorization of the 73 female sterility candidate genes of *T. reesei* QM6a. CAZymes: carbohydrate active enzymes, which include glycosyl hydrolases, glycosyl transferases and accessory enzymes. The corresponding FunCat numbers are: 01, metabolism; 01.25.01, CAZymes; 11.02.03.04, transcription factors; 20.01, solute transport; 01.25.03, proteases; 30, signal transduction; 99, protein without described function, i.e., orphan (if the encoded protein has no homolog outside the genus *Trichoderma*), or unknown (if the encoded protein has orthologs in other species than *Trichoderma*). “Others” comprise genes that do not fall into any of the listed categories

Whole genome comparison of *T. reesei* RL1/A8-02, RL2/A8-11 and QM6a (Tables 1, 2) revealed that strain RL1/A8-02 shows a higher degree of similarity with the parental strain QM6a than strain RL2/A8-11, as both the number of SNVs and DIPs (deletion/insertion polymorphism) is smaller. We identified a total number of 427 genes, in which a SNV or DIP caused a change of the amino acid sequence of the encoded protein in QM6a. The actual number of affected genes is lower than the sum of SNVs and DIPs (497) since most genes carrying a DIP are also affected by SNV(s). To increase the likelihood of tracking genes causing female sterility in strain QM6a, we only focused on those SNVs and DIPs which the two inbred strains had in common and which differentiated these strains from strain QM6a. Out of these, we selected those genes in which the SNVs and DIPs resulted in a change of the amino acid sequence of the corresponding proteins, excluded those genes in which SNVs and DIPs resulted in the occurrence of a premature stop codon in strains RL1/A8-02 and RL2/A8-11, and excluded all genes in which SNVs resulted in a conserved amino acid exchange (e.g., A- > V, I- > L, K- > E, etc.). For the remaining genes a BLASTX search was performed against the NCBI database to exclude all genes for which the amino acid changes in the proteins were located in non-conserved regions. Using this approach, the number of candidates was reduced to 73 genes (Additional file 3: Table S2). The majority of them encoded unknown proteins that were conserved in other Pezizomycotina and enzymes of intermediary and secondary metabolism (Fig. 1). All the other gene groups (encoding carbohydrate active enzymes, solute transporters, transcription factors, proteases and components of signal transduction pathways) comprised only 3 (in one case 5) members.

**Table 1:** Summary of the occurrence of SNVs in the two inbred strains RL1/A8-02 and RL2/A8-11

	SNV/A8-02	%-02	SNV/A8-11	%-11	SNV/Com
Number of SNVs	77,403	56	118,600	36	43,282
Number of SNVs in annotated regions	25,390	59	40,042	37	14,999
Number of SNVs in coding regions	23,987	59	37,484	38	14,199
Genes with SNVs in coding regions	1060	60	1687	38	638
Number of amino acid change(s)	4806	65	7044	44	3124
Genes with amino acid change(s)	746	57	1127	38	427

*SNV/A8-02* SNVs occurring in strain RL1/A8-02, *SNV/A8-11* SNVs occurring in strain RL2/A8-11, *SNV-Com* SNVs co-occurring in both inbred strains, *%-02* common SNVs in percentage of the SNVs of strain RL1/A8-02, *%-11* common SNVs in percentage of the SNVs of strain RL2/A8-11

**Table 2:** Summary of the occurrence of DIPs in the two inbred strains RL1/A8-02 and RL2/A8-11

	DIP/A8-02	%-02	DIP/A8-11	%-11	DIP/Com
Number of DIPs	8945	53	15,378	31	4751
Number of DIPs in annotated regions	1578	53	2920	28	829
Number of DIPS in coding regions	1293	51	2348	28	661
Genes with DIPs in coding regions	535	55	907	32	293
Number of amino acid change(s)	279	54	512	30	151
Genes with amino acid change(s)	136	52	250	28	70

*DIP/A8-02* DIPs occurring in strain RL1/A8-02, *DIP/A8-11* DIPs occurring in strain RL2/A8-11, *DIP-Com* DIPs co-occurring in both inbred strains, *%-02* common DIPs in percentage of the DIPs of strain RL1/A8-02, *%-11* common DIPs in percentage of the DIPs of strain RL2/A8-11

### Most of the sequence differences are clustered on the mating type chromosome

**Fig. 2:**
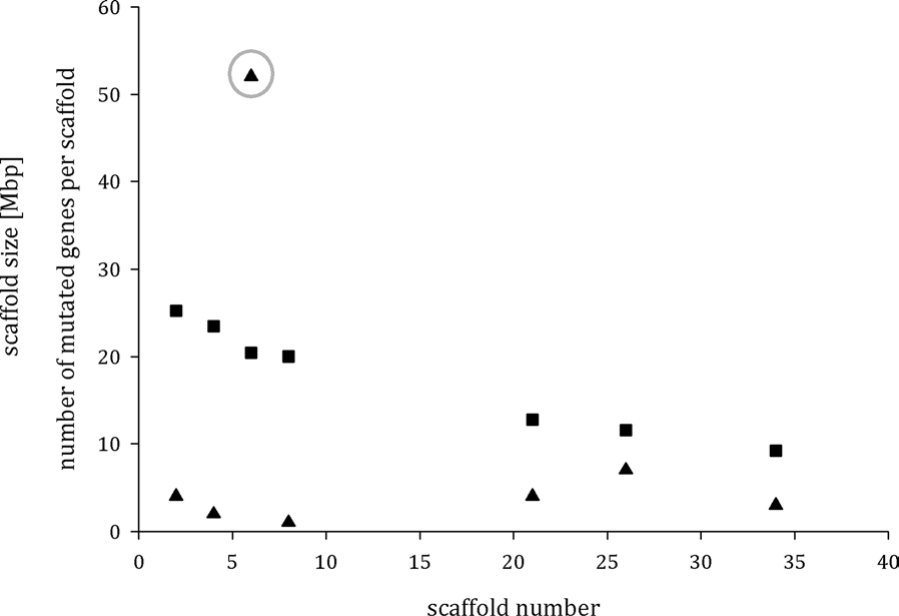
Plot of scaffold size (*full squares*) and the selected 73 genes differing in their sequence from *T. reesei* QM6a on the respective scaffolds (*full triangles*) vs. scaffold number. The *gray circle* around the *triangle* corresponds to scaffold 6 and notes the in relation to scaffold size disproportionately high number of genes differing in their sequence from strain *T. reesei* QM6a

An intriguing feature, which emerged during the functional annotation of the 73 genes, was that their genomic location was strongly biased: the majority of them (52) were located on scaffold 6 (Additional file 3: Table S2; Fig. 2) which harbors the mating type locus and an over proportionally high number (4 and 7) were located on scaffolds 21 and 26, respectively. A recently released computational model of the assembled *T. reesei* chromosomes [18] suggests that these scaffolds are not located on the same chromosome which corroborates our data that scaffold 6, 21 and 26 do not reside in direct neighborhood (Linke et al. unpublished data).

### Identification of genes involved in mating of *T. reesei*

For the identification of those genes among the 73 candidates (vide supra) that were crucial for mating, we systematically produced gene knock-outs in a *tku70* deleted derivative of *T. reesei* strain RL1/A8-02 and tested whether the resulting deletion strains are still capable of mating and fruiting body formation with QM6a (*MAT1*-*2*). Deletion of 2 of the 73 genes—Trire2:67350 and Trire2:59270—consistently resulted in a fruiting-body deficient phenotype. Their reintroduction into the respective knock-out strain restored the mating competence and fruiting body formation. For three genes Trire2:3422, Trire2:47930 and Trire2:81593 we were not able to obtain a deletion strain.

**Fig. 3:**
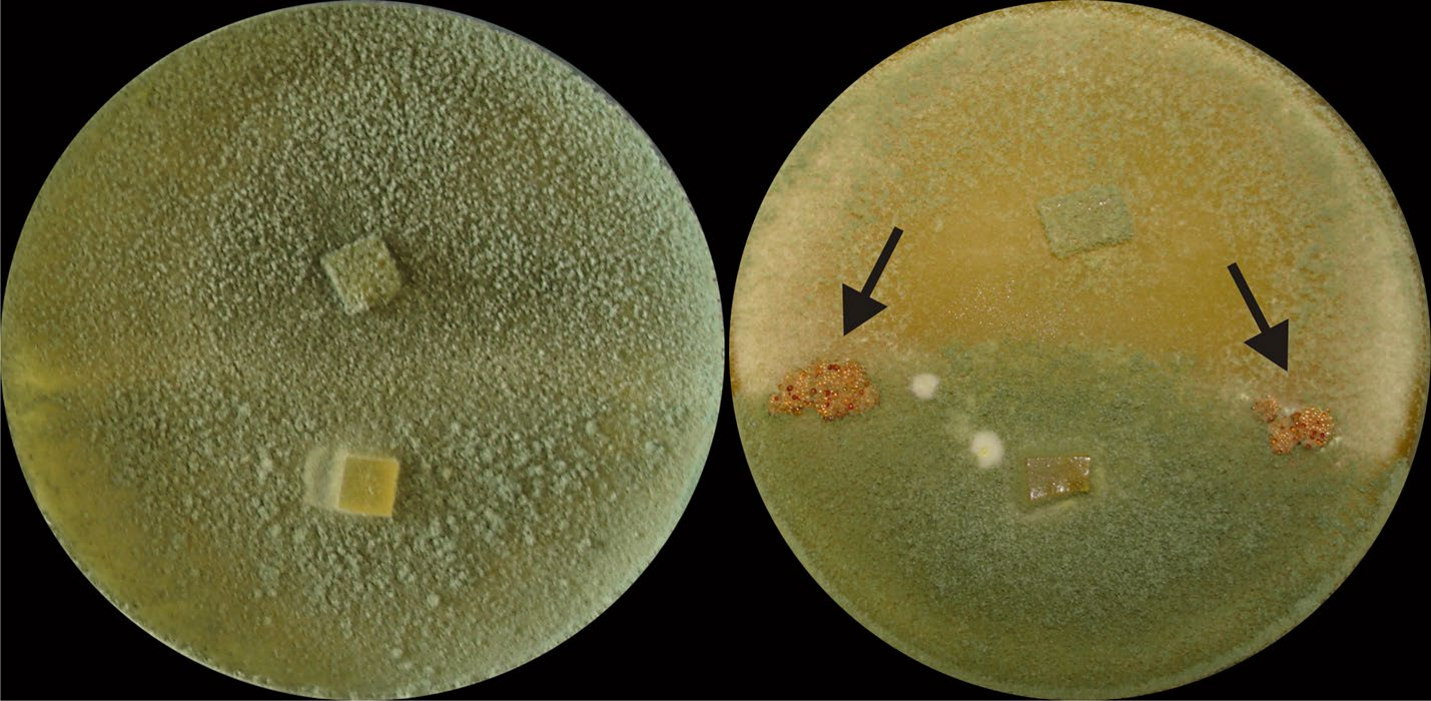
Mating assays of *T. reesei* QM6a *MAT1*-*2* with a mating type switched *T. reesei* QM6a *MAT1*-*1* (*left*) and with a mating type switched *T. reesei* QM6a *MAT1*-*1* which was additionally complemented with the functional *ham5* allele of CBS999.97 (*right*). Due to the effect of female sterility no fruiting bodies are formed in the contact zone of a QM6a *MAT1*-*2* strain with a mating type switched QM6a. After complementation with a functional *ham5* allele the phenotype is altered, sporulation is reduced and fruiting bodies (indicated by *black arrows*) appear in the contact zone of the two strains

To test whether any of these five genes are associated with female sterility of *T. reesei* QM6a we first constructed a recombinant QM6a *MAT1*-*1* strain by replacing the *MAT1*-*2* locus with the *MAT1*-*1* locus of strain C.P.K. 1282. The integration of the respective genes of the *MAT1*-*1* locus, *MAT1*-*1*-*1*, *MAT1*-*1*-*2* and *MAT1*-*1*-*3,* into the chromosomal DNA of mitotically stable transformants was confirmed by diagnostic PCR analysis (Additional file 4: Figure S2). Mating assays with other (non QM6a) *MAT1*-*2* wild-type strains confirmed that the strain is able to reproduce sexually with these strains. As expected from earlier results [12], this strain could not mate with QM6a. It was subsequently used as recipient strain for complementation with the five genes (the two genes whose knockout resulted in a fruiting body deficient phenotype and those for which no deletion strains could be obtained). The CBS999.97 alleles of the single genes as well as different combinations of them were transformed into QM6a *MAT1*-*1*. Single spore isolated transformants were then tested for their ability to produce fruiting bodies with the *MAT1*-*2* wild-type isolate QM6a. A diagnostic PCR analysis of the fertile transformants identified the CBS999.97 Trire2:67350 allele to be responsible for the recovery of female fertility in a QM6a *MAT1*-*1* strain as this allele was present in all mating positive transformants recorded (Fig. 3).

### Trire2:67350 is the orthologue of *N. crassa* HAM-5 and *P. anserina* IDC1

**Fig. 4:**
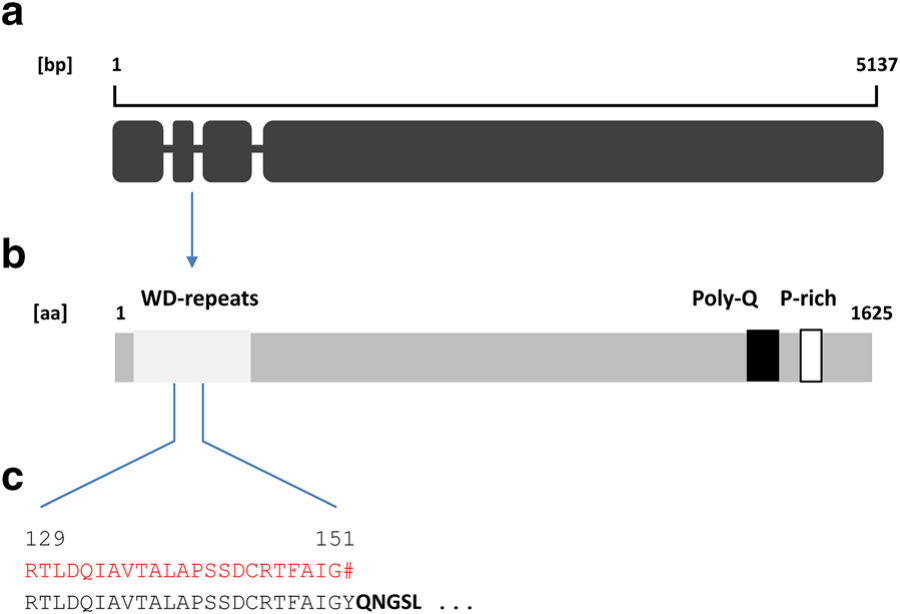
The *ham5* locus (**a**), the in silico translated and cDNA-confirmed HAM5 protein (**b**), and a comparison of the amino acid sequence of HAM5 (**c**) of *T. reesei* QM6a (*red*) and of *T. reesei* RL2/A8-02 (*black*). The *vertical lines* serve to relate the respective regions in **a**, **b** and **c**

Trire2:67350 was originally annotated in the *T. reesei* genome database v2.0 (http://www.genome.jgipsf.org/Trire2/Trire2.home.html) as a hypothetical protein and the corresponding gene harbors five introns. Its sequence is moderately conserved among Ascomycetes (Additional file 5: Table S3). In order to compare the amino acid sequence of the functional allele of Trire2:67350 with that of its orthologs from other fungi, we isolated RNA and sequenced the corresponding cDNA from strains RL1/A8-02 and RL2/A8-11. This showed that the *T. reesei* gene is 5121 nt long and interrupted by three short introns residing in the 5′-terminal fifth of its sequence (Fig. 4a). In silico translation yields a 1625 amino acids protein with a Mw of 178.133 kDa. Using this sequence its orthologs were identified as *ham*-*5* (*Neurospora crassa*) and *idc1* (*Podospora anserina*). The *T. reesei* HAM5 protein, similar to its orthologs in other Pezizomycota, carries a serine-rich WD40 repeat (IPR015943) region between amino acids 40 and 320, suggesting a possible protein–protein interaction site. In addition, and also consistent with its orthologs in other fungi, the protein exhibits a proline-rich region (C-terminal) which is also conserved among different species. Interestingly, the *T. reesei* HAM5 contains an additional stretch of glutamine residues (35 of 38 residues) close to the proline-rich region (Fig. 4b). This polyglutamine stretch is also present in *T. virens,* although much shorter than that in *T. reesei* and half of it replaced by a proline repeat. In *T. atroviride* it is even shorter and flanked by glutaminic acid repeats at its N-terminal side and proline repeats on its C-terminal side (Additional file 6: Figure S4). However, the polyglutamine stretch is completely absent in other fungal species even in phylogenetically close ones such as *Beauveria spp.* or *Metarhizium spp*.

To identify the mutation that renders *ham5* in *T. reesei* QM6a non-functional, we compared its genomic sequence with that of strains RL1/A8-02 and RL2/A8-11. The *ham5* gene of *T. reesei* QM6a differed in a total of 91 nucleotide positions of which 9 occurred in introns (Additional file 7: Figure S3). One of them (G_531_ → T) occurred at the first position of the second intron, thus changing the invariant G_+1_ of the donor splicing site [19] to a T. Sequencing of the cDNA formed by strain QM6a confirmed that as a consequence of this mutation, this second intron is not spliced, which results—due to the subsequent in frame occurrence of a TAA stop codon formed by the internal donor splicing site (5′-TTAAGT-3′)—in the formation of a truncated protein of only 151 amino acids in *T. reesei* QM6a (Fig. 4c).

## Discussion

In this study, we have identified the genetic basis for the female sterility of *T. reesei* QM6a [12] and strains derived from it. The availability of mating competent *T. reesei* QM6a derived strains now allows, as shown here, crossing of strains from relevant lineages of *T. reesei* to combine industrially important genetic traits, and thus to include strain crossing in industrial strain improvement programs.

Strain crossing has only rarely been used with filamentous fungi [cf. 5], probably due to the fact that most of the respective fungi were isolated as asexual individuals or rapidly lost the ability for sexual development upon cultivation in the laboratory. Fungal field populations have a female-sterile/hermaphrodite status and female-sterile mutants may comprise >50 % of the population [20]. Saleh et al. [21] have shown that in *Magnaporthe oryzae* asexual propagation results in loss of sexual reproduction after only a couple of generations. They interpreted this loss as the result of genetic drift after the introduction of the fungus into a new environment. This is essentially what happens when a fungus is purified from an environmental sample and maintained in a laboratory environment. We, therefore, expect that loss of sexual propagation may also have occurred with most fungi used in biotechnology. In fact, recent success in crossing of industrially applied strains of *P. chrysogenum* also revealed strains that failed to recombine [7], and thus these may also have acquired mutations in genes essential for sexual reproduction. Therefore, the strategy used in this paper may stimulate respective research also in other industrial fungi.

One gene that was identified in this study as the cause for the observed female sterility in *T. reesei* QM6a is highly conserved in Ascomycetes and an ortholog of the recently identified *P. anserinaidc1* and *N. crassa ham*-*5* [22, 23]. The loss of function of this gene has been reported in *N. crassa* to result in defective conidial anastomosis tube formation and impairment of hyphal fusion during colony initiation and in the mature colony [22, 24]. In addition it caused a lack of formation of pigment and aerial hyphae, female sterility, and the inability to trigger cell degeneration in *P. anserina* [23]. The above-described cell fusion that occurs between vegetative cells as a prerequisite for colony establishment is also required for mating [25–27], and thus several *N. crassa* mutants deficient in hyphal fusion and/or signaling are also female sterile [27]. Therefore, our identification of *ham5* as a gene defective in *T. reesei* QM6a and being responsible for its female sterility agrees well with these findings.

While this paper was prepared for submission, HAM5 was shown to physically interact with a conserved MAP kinase cascade that includes NRC-1, MEK-2 and MAK-2, suggesting that it functions as a scaffold/transport hub for the MAP kinase cascade members for oscillation and chemotropic interactions during germling and hyphal fusion in *N. crassa* [28]. This finding is further supported by Dettmann et al. [29] who just recently identified HAM5 as a cell–cell communication-specific scaffold protein of the *Neurospora crassa* MAK-2 cascade (homologous to the budding yeast pheromone pathway).

The most striking difference in the protein sequence of *T. reesei* HAM5 from that of other Ascomycetes is the occurrence of the polyglutamine stretch in *T. reesei* which is lacking in other fungal genera. Since the nucleotide sequence encoding this polyglutamine repeat is not replaced by an intron in other fungi, it is important to note that the apparent lack of occurrence of the polyglutamine stretch in these fungi is not due to a potentially wrong intron prediction. The function of polyglutamine sequences is still under debate. One hypothesis postulates that they simply form a flexible spacer between protein domains [30]. On the other hand, polyglutamine sequences are generally believed to mediate protein–protein interaction [31], e.g., in transcription factors [32], where they can activate gene transcription [33, 34] and nuclear localization [35, 36]. Schaefer et al. [30] also reported that polyglutamine tracts are often accompanied by proline-rich regions in their C-terminal vicinity, which is the case in *T. reesei* as well. It is also intriguing to note that the polyglutamine stretch is longest in *T. reesei* and shortest in *T. atroviride*. The latter is phylogenetically more ancient than *T. reesei* [37] which lends to speculate that the polyglutamine stretch in HAM5 arose during the evolution of *Trichoderma*. Whether this endowed HAM5 with genus-specific functions not present in other fungi, or simply served to adapt its function to *Trichoderma* physiology will be an intriguing subject for further studies.

## Conclusion

The discovery of the sexual life cycle of *T. reesei* by Seidl et al. [12] was the first step towards the application of classical strain breeding in *T. reesei*. The identification of the strain’s female sterility, however, precluded the use of classical genetic tools for strain improvement. By identifying the cause for female sterility on a genetic basis we have now provided the basis for the use of strain improvement by sexual crossing in *T. reesei*. To the best of our knowledge, we here provide the first example where advanced sequencing technologies in combination with a back-crossing strategy and a systematic knockout program allowed to identify relevant mutations in an industrial fungus. We strongly believe that the strategy used in this paper can be applied to resolve many other unclear genotype phenotype correlations and so help to overcome bottlenecks in further improvement of strains.

## Methods

### Strains and cultivations

The strains used throughout this work are listed in Additional file 1: Table S1. Strains were cultivated on potato dextrose agar (PDA; Difco, Sparks, MD, USA) and maintained on 28 °C in darkness. Crossing of strains was performed by opposing the two mating partners on a PDA plate on the benchtop (temperature variations between 18 and 22 °C under day/night light fluctuations) for 10–14 days until formation of fruiting bodies and ascospore discharge could be detected.

### Construction of inbred strains RL1/A8-02 and RL2/A8-11

Sexual crossings of QM6a (*MAT1*-*2*) with strain CBS999.97 (*MAT1*-*1*) were performed followed by repeated cycles of backcrossing of the *MAT1*-*1* progenies with QM6a (*MAT1*-*2*). Fruiting bodies ejected their ascospores to the cover of the Petri dish from where they were collected. The mating type of several single spore isolated offsprings was tested by diagnostic PCR (sequence of oligonucleotides is given in Additional file 8: Table S4). Approximately 5 *MAT1*-*1* carrying individuals were then used for a mating assay to produce another generation of offsprings. By this method, we could obtain fertile strains carrying the *MAT1*-*1* locus with a maximum of QM6a-specific genes. The resultant progenies from the eighth generation were then tested for their ability to mate with *T. reesei* QM6a and other *MAT1*-*2* strains. One strain of two independent inbred lines each (RL1/A8-02 and RL2/A8-11) was then used for genome sequencing.

### Genome sequencing of *T. reesei* RL1/A8-02 MAT1-1 *and* RL2/A8-11 MAT1-1

Two libraries, with 320 bp and 8 kbp insert size, were prepared for each strain, respectively. To this end, DNA was fragmented using a Covaris S2 system (Covaris, Inc. Woburn, MA, USA) and fragments were purified using the QIAquick PCR purification kit (Qiagen, Hilden, Germany). Paired-end libraries were prepared using the NEBNext DNA Sample Prep modules (New England Biolabs, Ipswich, MA, USA) following the manufacturer’s instructions. Briefly, fragments were end-repaired using Klenow and T4 DNA polymerases and phosphorylated with T4 polynucleotide kinase. Fragments were then 3′-adenylated using Klenow exo-DNA polymerase, and Illumina adapters were added using DNA ligase. Ligation products of ~400 bp were gel-purified using the Qiagen gel extraction kit (Qiagen, Hilden, Germany). To avoid the introduction of a guanine–cytosine (GC) bias during the gel-purification step in the standard Illumina library preparation protocol, the gel slice was dissolved rather at room temperature than with heating. The size-selected, adapter-modified DNA fragments were PCR amplified using PE PCR primers 1.0 and 2.0 (Illumina, San Diego, CA, USA) using Phusion DNA polymerase (New England Biolabs, Ipswich, MA, USA) and the following protocol: polymerase activation (98 °C for 30 s), followed by 10 cycles (denaturation at 98 °C for 10 s, annealing at 65 °C for 30 s and extension at 72 °C for 50 s) with a final, 5-min extension at 72 °C. Libraries were purified and quantified using the Qubit HS Assay Kit (Invitrogen, Carlsbad, CA, USA).

Cluster amplification was performed using the TruSeq PE Cluster Kit v5 on a cluster station, and all libraries were sequenced on a single Illumina HiSeq 2000 lane using TruSeq SBS 36 Cycle Kits v5 (Illumina, San Diego, CA, USA) using a 2 × 107 bp paired-end protocol. Sequencing image files were processed using Illumina’s Sequencing Control Software (SCS) by Illumina’s Real Time Analysis (RTA) v2.6 and CASAVA v1.7 (Illumina Inc., San Diego, CA, USA) to generate base calls and Phred-like base quality scores and to remove failed reads.

Sequences were quality filtered using CLC Genomic Workbench (version 5.1, CLC bio, Arhus, Denmark) and de novo assembled with Newbler (version 2.60, Roche/454, Brandford, CT, USA) and CLC Genomic Workbench. To identify large insertions in the two backcrossed genomes which are not present in the published *T. reesei* QM6a genome sequence, the obtained contigs were mapped to the scaffolds of *T. reesei* QM6a (accessions: GL985056-GL985132; NC_003388) using BLAST [38] and r2cat [39]. The BLAST hits were subsequently processed with a custom R script. Single nucleotide variation (SNVs) and insertions and deletions (DIPs) between the QM6a reference sequence and the aligned sequences of the two backcrossed lines were identified using CLC Genomic Workbench. Candidate genes were then manually tested for silent mutations. These and sequence differences that lead to a conserved amino acid exchange that does not interfere with the function of the putative protein were excluded from further analysis and discarded.

Genes were then classified according to their annotation in the *T. reesei* genome database v2.0 (Joint Genome Institute, http://www.genome.jgi.doe.gov, [40]), the MIPS Functional Catalogue (http://www.mips.helmholtz-muenchen.de/proj/funcatDB, [41]) and a manually curated completely annotated genome database of *T.* *reesei* present proprietary to the authors at TU Vienna.

### Data deposition

The sequences reported in this paper have been deposited in the EMBL-EBI short read archive under the accession number ERP009132. The *T. reesei* HAM5 protein sequence derived from cDNA sequencing is available in GenBank with accession number KM677955.

### Fungal strain constructions

*Trichoderma reesei* was transformed via protoplast transformation [42]. Deletion cassettes consisting of 1.0–1.5 kb fragments of gene-specific flanking regions interrupted by the selection marker were assembled by yeast recombinational cloning as described in detail by Schuster et al. [43]. Individual flanking regions of candidate genes were amplified using oligonucleotides 5F + 5R as well as 3F + 3R from genomic DNA of strain CBS999.97 with Phusion Polymerase (Thermo Fisher Scientific/Fermentas, St. Leon-Rot, Germany). As marker the *hph* expression cassette (2.7 kb) was amplified by PCR from the plasmid pLHhph [44] using the oligonucleotides hph fw and hph rv. All sequences of the oligonucleotides used are given in Additional file 8: Table S4.

To construct a *T. reesei* QM6a *MAT1*-*1* strain, the resident *MAT1*-*2* locus was replaced by the *MAT1*-*1* locus from *T. reesei* C.P.K. 1282. A 5500 bp fragment of this *MAT1*-*1* locus, including additional 3300-bp upstream and 1300-bp downstream sequences was amplified from genomic DNA using the primers MAT1-1_fw and MAT1-1_rv (Additional file 8: Table S4), and ligated into vector pCR blunt (Life Technologies/Invitrogen; Carlsbad, CA, USA). The *hph* selection cassette [44] was cloned into the *Avr*II site, situated in the intergenic region between *MAT1*-*1*-*3* and Trire2:59147 (encoding a DNA lyase). For transformation of *T. reesei* QM6a, the replacement cassette was PCR amplified using the primer pair 1-2_replace_cassette_fw and 1-2_replace_cassette_rv (Additional file 8: Table S4). Strains were tested for integration of the cassette by PCR and mitotic stability. Positive strains were further tested for their ability to mate with *MAT1*-*2* wild-type isolates.

Strain QM6a (*MAT1*-*1*) was complemented with the five putatively functional alleles of genes Trire2:67350, Trire59270, Trire2:3422, Trire2:47930 and Trire2:81593 of strain CBS999.97 (*MAT1*-*1*). The genes were PCR amplified (sequences of the oligonucleotides are given in Additional file 8: Table S4) and ligated/cloned by In-Fusion recombination (Clontech, Mountainview, CA, USA) in a derivative of pPki-Gen [45] where the *pki1* promoter was removed by an *Eco*RI and *Xba*I digest (Thermo Fisher Scientific/Fermentas, St. Leon-Rot, Germany). Transformants were selected and purified on PDA plates containing 100 µg/ml geneticindisulfate G418 (Carl Roth GmbH + Co. KG, Karlsruhe, Germany). A scheme of the vector construct to complement strain QM6a *MAT1*-*1* with the 5 final candidate genes is given in Additional file 9: Figure S5. The vector construction for the complementation with the functional allele of Trire2:67350 (pFSG_ID67350) serves as an example since all plasmids were constructed in the same manner. The integration of the five respective genes was verified by PCR using each a gene specific primer and a second primer binding within the selection marker to exclusively screen for the integration of the CBS999.97 specific allele. Sequences of the oligonucleotides used for PCR analysis are given in Additional file 8: Table S4.

### RNA extraction and cDNA synthesis of Trire2:67350

To obtain cDNAs of *ham5* for determining the respective protein sequence of *T. reesei* QM6a, RL1/A8-02 and RL2/A8-11, strains were cultivated on PDA plates covered with cellophane. Total RNA was isolated according to Gruber et al. [46]. Briefly, mycelium was ground in liquid nitrogen and transferred to reaction tubes containing a mixture of 650 µl lysis buffer (0.6 M NaCl, 10 mM EDTA, 100 mM Tris pH 8 and 4 % SDS) and 650 µl phenol (pH 8). Samples were vortexed well and placed on ice for 15 min. Thereafter, samples were centrifuged at >13,000 rpm at 4 °C for 20 min. The supernatant was transferred to a fresh reaction tube containing 8 M LiCl, vortexed well and put on 4 °C overnight. The samples were centrifuged again at >13,000 rpm at 4 °C for 20 min and the pellet was dissolved in 750 µl isopropanol. For precipitation the samples were placed at −20 °C for 2 h, then centrifuged at >13,000 rpm at 4 °C for 20 min. The RNA pellet was finally dissolved in deionized water.

DNase treated (DNase I, RNase free; Thermo Fisher Scientific/Fermentas, St. Leon-Rot, Germany) RNA (5 µg) was reverse transcribed with the RevertAid™ First Strand cDNA Kit (Thermo Fisher Scientific/Fermentas, St. Leon-Rot, Germany) according to the manufacturer’s protocol with a combination of the provided oligo-dT and random hexamer primers.

## Additional files

Additional file 1:
**Table S1.** List of strains used in and constructed within this study. Additional file 2:
**Figure S1.** Pedigree of the *T. reesei* inbred strains RL1/A8-02 and RL2/A8-11 subjected to genome sequencing. “X” denotes crossing. Additional file 3:
**Table S2.** Genes bearing sequence differences in *T. reesei* QM6a when compared to *T. reesei* RL1/A8-02 and RL2/A8-11 and selected for the gene knockout program to identify the cause of female sterility in *T. reesei* QM6a. Additional file 4:
**Figure S2.** PCR confirmation of the exchange of the *MAT1*-*2* locus in strain *T. reesei* QM6a by the *MAT1*-*1* idiomorph amplified from strain C.P.K. 1282. The longer amplicon corresponds to the *MAT1*-*1*-*2* gene and the shorter one to the *MAT1*-*2*-*1* gene. To ascertain these results the strain QM6a (*MAT1*-*1*) was further tested in mating assays using strain CBS999.97 (*MAT1*-*2*) as mating partner. Additional file 5:
**Table S3.** Fifty best BLAST hits (NCBI) for Trire2:67350 using the protein sequence of RL1/A8-02 derived from cDNA sequencing. Additional file 6:
**Figure S4.** Alignment of the polyglutamine tracts from *T. reesei* QM6a with that of the orthologs of *T. virens* (Trive2:50286) and *T. atroviride* (Triat2:221851). Additional file 7:
**Figure S3.** Nucleotide sequence of the *ham5* locus from *T. reesei* QM6a and *T. reesei* RL1/A8-02 including the mutation being critical for female fertility. The sequence differences are marked in gray, introns are underlined. The critical mutation (G- > T) in the second intron of strain QM6a is indicated by a red box and the resulting stop codon is labeled in yellow. Additional file 8:
**Table S4.** Sequences of the primers used for mating-type determination, the vector construction for the direct MAT locus replacement in *T. reesei* QM6a *MAT1-2*, the construction of the deletion cassettes for Trire2:59270 and Trire2:67350 and the plasmid construction for gene complementation in *T. reesei* QM6a *MAT1-1*. Additional file 9:
**Figure S5.** Schematic drawing of the construction of the plasmid for the complementation of *T. reesei* QM6a with the wild-type allele of Trire2:67350 (*ham5*).

## Abbreviations

DIP: deletion/insertion polymorphism
*MAT1*-*1*: mating type 1-1
*MAT1*-*2*: mating type 1-2
SNV: single nucleotide variant
*T. reesei*: *Trichoderma reesei*
