# Supplementary material for: Restoration of female fertility in Trichoderma reesei QM6a provides the basis for inbreeding in this industrial cellulase producing fungus
Source: Biotechnol Biofuels. 2015 Sep 24;8:155. doi: 10.1186/s13068-015-0311-2 (PMC4581161; doi:10.1186/s13068-015-0311-2)
Supplement: Additional file 1: — Table S1. List of strains used in and constructed within this study. [file 13068_2015_311_MOESM1_ESM.docx]

**Supplementary Table S1:** List of strains used in and constructed within this study.

| **Strain** | **Mating type** | **Strain type** | **Mating competent with** |
| --- | --- | --- | --- |
| QM6a | *MAT1-2* | Wild-type | CBS999.97/*MAT1-1*, C.P.K. 1282, C.P.K. 170, RL1/A8-2, RL2/A8-11, RL1/A8-2 *Δtku70,* QM6a complemented with CBS Trire2:67350/*MAT1-1* |
| CBS999.97 | *MAT1-1* | Wild-type | QM6a/MAT*1-2*, CBS999.97/MAT*1-2*, C.P.K. 938, QM6a complemented with CBS Trire2:67350/*MAT1-2* |
| CBS999.97 | *MAT1-2* | Wild-type | CBS999.97/*MAT1-1*, C.P.K. 1282, C.P.K. 170, RL1/A8-2, RL2/A8-11, RL1/A8-2 *Δtku70,* RL1/A8-2 *Δtku70ΔTrire2:67350,* QM6a complemented with CBS Trire2:67350/*MAT1-1* |
| C.P.K. 1282 (G.J.S. 85–249) | *MAT1-1* | Wild-type | QM6a/*MAT1-2*, CBS999.97/MAT*1-2*, C.P.K. 938, QM6a complemented with CBS Trire2:67350/*MAT1-2* |
| C.P.K. 170 (G.J.S. 86–410) | *MAT1-1* | Wild-type | QM6a/MAT*1-2*, CBS999.97/MAT*1-2*, C.P.K. 938, QM6a complemented with CBS Trire2:67350/*MAT1-2* |
| C.P.K. 938 (G.J.S. 89-7) | *MAT1-2* | Wild-type | CBS999.97/*MAT1-1*, C.P.K. 1282, C.P.K. 170, RL1/A8-2, RL2/A8-11, RL1/A8-2 *Δtku70,* QM6a complemented with CBS Trire2:67350/*MAT1-1* |
| RL1/A8-02 | *MAT1-1* | Inbred strain | QM6a/MAT*1-2*, CBS999.97/MAT*1-2*, C.P.K. 938, QM6a complemented with CBS Trire2:67350/*MAT1-2* |
| RL2/A8-11 | *MAT1-1* | Inbred strain | QM6a/MAT*1-2*, CBS999.97/MAT*1-2*, C.P.K. 938, QM6a complemented with CBS Trire2:67350/*MAT1-2* |
| RL1/A8-02 *Δtku70* | *MAT1-1* | Recombinant | QM6a/MAT*1-2*, CBS999.97/MAT*1-2*, C.P.K. 938, QM6a complemented with CBS Trire2:67350/*MAT1-2* |
| RL1/A8-02 *Δtku70ΔTrire2:67350* | *MAT1-1* | Recombinant | CBS999.97/MAT*1-2*, C.P.K. 938, QM6a complemented with CBS Trire2:67350/*MAT1-2* |
| QM6a | *MAT1-1* | Recombinant | CBS999.97/MAT*1-2*, C.P.K. 938, QM6a complemented with CBS Trire2:67350/*MAT1-2* |
| QM6a complemented with CBS Trire2:67350 | *MAT1-1* | Recombinant | QM6a/MAT*1-2*, CBS999.97/MAT*1-2*, C.P.K. 938, QM6a complemented with CBS Trire2:67350/*MAT1-2* |
| QM6a complemented with CBS Trire2:67350 | *MAT1-2* | Recombinant | CBS999.97/*MAT1-1*, C.P.K. 1282, C.P.K. 170, RL1/A8-2, RL2/A8-11, RL1/A8-2 *Δtku70,* RL1/A8-2 *Δtku70ΔTrire2:67350,* QM6a complemented with CBS Trire2:67350/*MAT1-1* |
