# Supplementary figures and images for: Restoration of female fertility in Trichoderma reesei QM6a provides the basis for inbreeding in this industrial cellulase producing fungus
Source: Biotechnol Biofuels. 2015 Sep 24;8:155. doi: 10.1186/s13068-015-0311-2 (PMC4581161; doi:10.1186/s13068-015-0311-2)

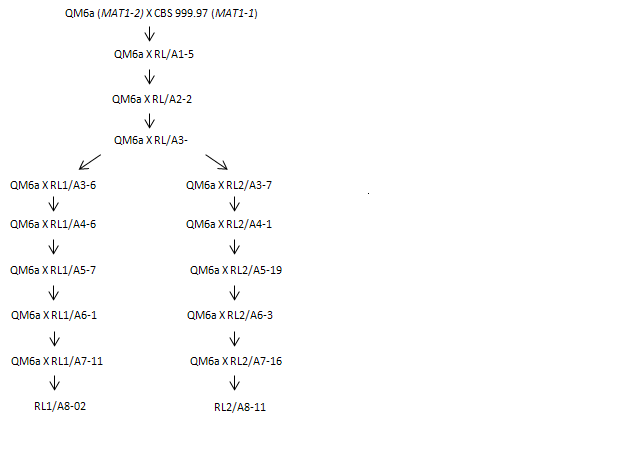

Supplement: Additional file 2: — Figure S1. Pedigree of the T. reesei inbred strains RL1/A8-02 and RL2/A8-11 subjected to genome sequencing. “X” denotes crossing. [file 13068_2015_311_MOESM2_ESM.tif]

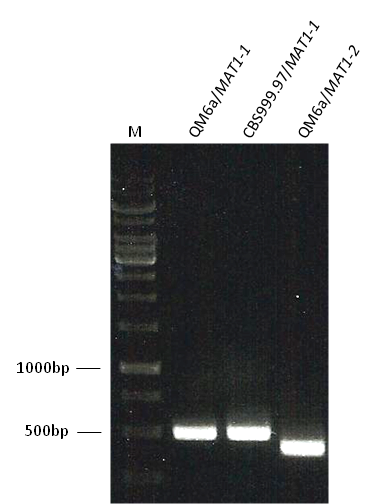

Supplement: Additional file 4: — Figure S2. PCR confirmation of the exchange of the MAT1-2 locus in strain T. reesei QM6a by the MAT1-1 idiomorph amplified from strain C.P.K. 1282. The longer amplicon corresponds to the MAT1-1-2 gene and the shorter one to the MAT1-2-1 gene. To ascertain these results the strain QM6a (MAT1-1) was further tested in mating assays using strain CBS999.97 (MAT1-2) as mating partner. [file 13068_2015_311_MOESM4_ESM.tif]

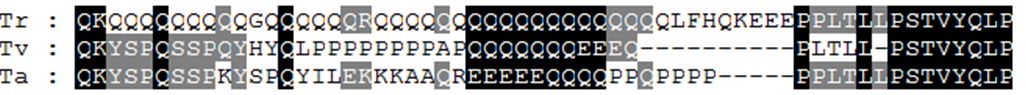

Supplement: Additional file 6: — Figure S4. Alignment of the polyglutamine tracts from T. reesei QM6a with that of the orthologs of T. virens (Trive2:50286) and T. atroviride (Triat2:221851). [file 13068_2015_311_MOESM6_ESM.tif]

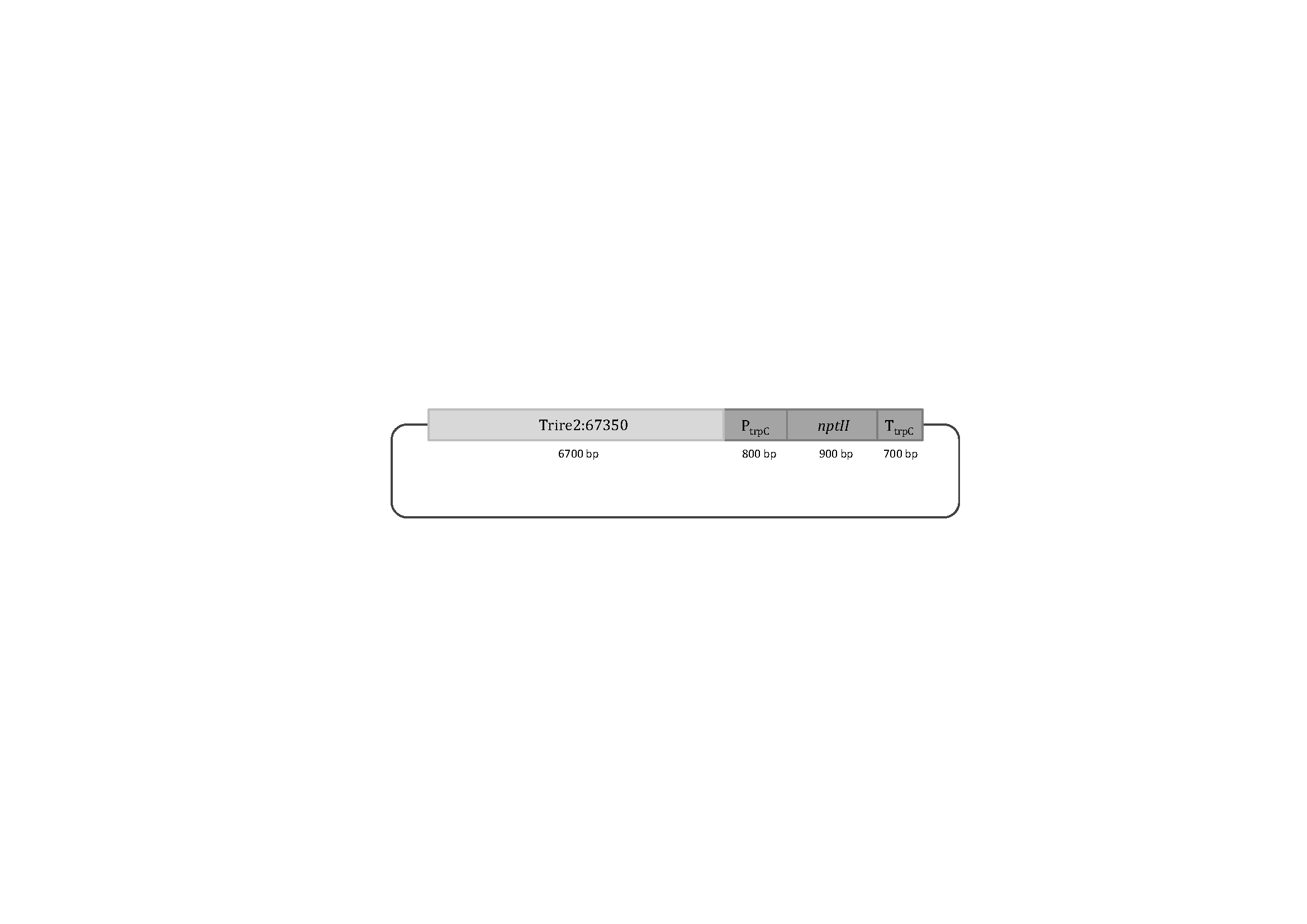

Supplement: Additional file 9: — Figure S5. Schematic drawing of the construction of the plasmid for the complementation of T. reesei QM6a with the wild-type allele of Trire2:67350 (ham5). [file 13068_2015_311_MOESM9_ESM.tif]
