# Supplementary material for: Restoration of female fertility in Trichoderma reesei QM6a provides the basis for inbreeding in this industrial cellulase producing fungus
Source: Biotechnol Biofuels. 2015 Sep 24;8:155. doi: 10.1186/s13068-015-0311-2 (PMC4581161; doi:10.1186/s13068-015-0311-2)
Supplement: Additional file 3: — Table S2. Genes bearing sequence differences in T. reesei QM6a when compared to T. reesei RL1/A8-02 and RL2/A8-11 and selected for the gene knockout program to identify the cause of female sterility in T. reesei QM6a. [file 13068_2015_311_MOESM3_ESM.docx]

**Supplementary Table S2:** Genes bearing sequence differences in *T. reesei* QM6a when compared to *T. reesei* RL1/A8-02 and RL2/A8-11 and selected for the gene knockout program to identify the cause of female sterility in *T. reesei* QM6a.

| **NCBI acc. Nr.** | **Trire2:** | **annotation** | **scaffold** | **mutation** | **begin*** | **end** | **SNVs**** | **Indels**** |
| --- | --- | --- | --- | --- | --- | --- | --- | --- |
| EGR49696.1 | 3262 | succinate semialdehyde dehydrogenase (NADP) | 6 | SNV | 54499 | 56324 | 2 | - |
| EGR49756.1 | 3310 | C2H2 transcriptional regulator | 6 | SNV | 521540 | 522173 | 3 | - |
| EGR49998.1 | 3363 | unknown protein, 9 TM | 6 | SNV | 934255 | 935322 | 6 | - |
| EGR49850.1 | 3397 | unknown protein | 6 | SNV | 1291186 | 1293056 | 3 | - |
| EGR50043.1 | 3400 | unknown protein | 6 | SNV & DIP | 1319464 | 1321584 | 1 | 1 |
| EGR49876.1 | 3422 | oxidoreductase of unknown function | 6 | SNV | 1450504 | 1451910 | 3 | - |
| EGR44478.1 | 5924 | unknown protein | 34 | SNV | 133342 | 134348 | 3 | - |
| EGR51590.1 | 21412 | unknown protein | 2 | SNV | 44212 | 44859 | 11 | - |
| EGR49821.1 | 27554 | AA9 accessory (former GH61) polysaccharide monooxygenase | 6 | SNV | 953025 | 954074 | 2 | - |
| EGR49812.1 | 27992 | PTH11 GPCR | 6 | SNV | 916490 | 917632 | 9 | - |
| EGR49944.1 | 35726 | Subtilisin like protease | 6 | SNV | 504033 | 506795 | 15 | - |
| EGR49694.1 | 36822 | unknown protein | 6 | SNV | 46981 | 49379 | 4 | - |
| AAP57759.1 | 46816 | GH3 β-glucosidase CEL3d | 6 | SNV | 13220 | 15392 | 3 | - |
| EGR49047.1 | 47930 | Mitochondrial oxoglutarate/malate carrier proteins | 8 | SNV | 1378834 | 1379787 | 1 | - |
| EGR45930.1 | 51217 | sulfate transporter, putative | 21 | SNV | 425663 | 427071 | 2 | - |
| EGR45162.1 | 51868 | unknown protein | 26 | SNV | 99355 | 100576 | 3 | - |
| EGR51617.1 | 55213 | tRNA-specific adenosine deaminase | 2 | SNV & DIP | 204812 | 206278 | 17 | 2 |
| EGR49906.1 | 59188 | unknown protein | 6 | SNV | 232547 | 234154 | 4 | - |
| EGR49712.1 | 59190 | MFS permease | 6 | SNV | 158907 | 160576 | 4 | - |
| EGR50032.1 | 59270 | unknown protein | 6 | SNV | 1221021 | 1223138 | 5 | - |
| EGR49882.1 | 59315 | PKS | 6 | SNV | 34746 | 46569 | 16 | - |
| EGR49720.1 | 59338 | D-Alanine aminotransferase | 6 | SNV | 209272 | 210237 | 7 | - |
| EGR49897.1 | 59351 | 1-aminocyclopropane-1-carboxylate synthase | 6 | SNV & DIP | 161087 | 162637 | 14 | 1 |
| EGR49863.1 | 59364 | oligopeptide transporter ISP4 | 6 | SNV | 1367318 | 1369953 | 9 | - |
| EGR49943.1 | 59368 | unknown protein, Duf341 | 6 | SNV | 486801 | 487766 | 1 | - |
| EGR49925.1 | 59372 | unknown protein | 6 | SNV | 354842 | 355606 | 4 | - |
| EGR49814.1 | 59381 | SAM-dependent methyltransferases | 6 | SNV | 927919 | 929457 | 1 | - |
| EGR49895.1 | 59391 | GH27 α-galactosidase | 6 | SNV | 139746 | 141784 | 3 | - |
| EGR50026.1 | 59396 | unknown protein | 6 | SNV | 1184222 | 1185858 | 5 | - |
| EGR49704.1 | 59402 | arsenate reductase Arc2 | 6 | SNV | 108309 | 108896 | 13 | - |
| EGR49899.1 | 59558 | unknown protein | 6 | SNV | 174021 | 175382 | 12 | - |
| EGR49768.1 | 59578 | GH13 α-glucosidase | 6 | SNV | 600205 | 602001 | 11 | - |
| EGR49737.1 | 59582 | unknown protein | 6 | SNV & DIP | 322843 | 324091 | 11 | 2 |
| EGR49726.1 | 59665 | unknown protein | 6 | SNV | 236239 | 238500 | 16 | - |
| EGR49728.1 | 59669 | extracellular salicylate hydroxylase/monooxygenase, putative | 6 | SNV | 243160 | 244580 | 10 | - |
| EGR49708.1 | 59689 | GH2 β-mannosidase | 6 | SNV | 143494 | 146398 | 4 | - |
| EGR49916.1 | 59700 | short chain dehydrognease/reductase | 6 | SNV | 303091 | 304148 | 9 | - |
| EGR49723.1 | 59723 | dipeptidyl peptidase 5 | 6 | SNV | 218118 | 219848 | 13 | - |
| EGR49832.1 | 59740 | transcriptional regulator, unknown | 6 | SNV | 1130946 | 1132285 | 2 | - |
| EGR49993.1 | 59751 | Ribonucleases P/MRP protein subunit POP1 containing protein | 6 | SNV & DIP | 867976 | 870786 | 17 | 1 |
| EGR49738.1 | 59760 | Zn2Cys6 transcriptional regulator | 6 | SNV | 325019 | 327253 | 24 | - |
| EGR49880.1 | 59771 | UbiA prenyltransferase, putative | 6 | SNV | 28628 | 29590 | 3 | - |
| EGR45918.1 | 67350 | unknown protein | 21 | SNV | 310069 | 315205 | 8 | - |
| EGR45848.1 | 67470 | unknown protein | 21 | SNV | 412213 | 413802 | 11 | - |
| EGR45203.1 | 68889 | PDR-type ABC transporters | 26 | SNV | 400579 | 404664 | 13 | - |
| EGR44490.1 | 70251 | vacuolar protein-sorting machinery class E protein HSE1 | 34 | SNV | 58487 | 60722 | 3 | - |
| EGR49919.1 | 76690 | unknown protein | 6 | SNV | 328885 | 330909 | 6 | - |
| EGR49958.1 | 76758 | maltose permease | 6 | SNV | 596683 | 598448 | 3 | - |
| EGR50002.1 | 76852 | GH2 β-galactosidase/β-glucuronidase | 6 | SNV | 985207 | 987542 | 6 | - |
| EGR50010.1 | 76862 | palmitoyltransferase PFA5, putative | 6 | SNV | 1040511 | 1041949 | 1 | - |
| EGR49837.1 | 76887 | aspartyl protease | 6 | SNV & DIP | 1197099 | 1198577 | 4 | 1 |
| EGR45146.1 | 81576 | Assimilatory sulfite reductase,Alpha subunit | 26 | SNV | 8754 | 11906 | 5 | - |
| EGR45155.1 | 81593 | MFS permease | 26 | SNV | 60509 | 62332 | 8 | - |
| EGR51873.1 | 103470 | unknown protein | 2 | SNV | 191884 | 195437 | 8 | - |
| EGR50649.1 | 104898 | unknown protein | 4 | SNV | 853808 | 855112 | 3 | - |
| EGR49884.1 | 105804 | PKS | 6 | SNV | 62708 | 69477 | 8 | - |
| EGR49889.1 | 105816 | unknown protein | 6 | SNV | 109088 | 110590 | 17 | - |
| EGR49709.1 | 105832 | 1-(5-Phosphoribosyl)-5-amino-4-imidazole-carboxylate (AIR) carboxylase | 6 | SNV | 149561 | 151463 | 3 | - |
| EGR49719.1 | 105849 | Zn2Cys6 transcriptional regulator | 6 | SNV & DIP | 199669 | 202640 | 21 | 1 |
| EGR49908.1 | 105866 | unique protein | 6 | SNV | 244971 | 245775 | 7 | - |
| EGR49736.1 | 105884 | short chain dehydrogenase/reductase | 6 | SNV | 321198 | 322115 | 9 | - |
| EGR49924.1 | 105894 | unknown protein | 6 | SNV & DIP | 348004 | 348407 | 3 | 1 |
| EGR49942.1 | 105924 | GT1 ß-glycosyltransferase | 6 | SNV | 484124 | 485739 | 3 | - |
| EGR49754.1 | 105931 | GH20 N-acetyl-β-hexosaminidase | 6 | SNV | 509949 | 512211 | 17 | - |
| EGR49860.1 | 106164 | short chain dehydrogenase/reductase | 6 | SNV | 1352069 | 1353345 | 7 | - |
| EGR45928.1 | 110648 | MIZ zinc finger protein | 21 | SNV & DIP | [405046](http://genome.jgi-psf.org/cgi-bin/browserLoad?db=Trire2&position=scaffold_21:405046-408869) | 408869 | 78 | 5 |
| EGR45176.1 | 111374 | unique protein | 26 | SNV & DIP | [180299](http://genome.jgi-psf.org/cgi-bin/browserLoad?db=Trire2&position=scaffold_26:180299-181809) | 181809 | 71 | 6 |
| EGR45201.1 | 111418 | unknown protein | 26 | SNV | [389302](http://genome.jgi-psf.org/cgi-bin/browserLoad?db=Trire2&position=scaffold_26:389302-391100) | 391100 | 2 | - |
| EGR51588.1 | 119991 | pre-mRNA splicing factor cwc24 | 2 | SNV | 18341 | 19520 | 4 | - |
| EGR50721.1 | 120806 | Ca/calmodulin-binding protein CMK2 | 4 | SNV | 1441491 | 1445358 | 6 | - |
| EGR49714.1 | 121136 | unique protein | 6 | SNV | 167849 | 169386 | 3 | - |
| EGR45233.1 | 123786 | NRPS (peptaibol synthase) | 26 | SNV & DIP | 277047 | 327620 | 2014 | 10 |
| EGR44492.1 | 124104 | unknown protein | 34 | SNV | 68807 | 73697 | 24 | - |

* begin and end relate to the position on the scaffold

** only sequence differences are quoted which concern exons
