# Supplementary material for: Restoration of female fertility in Trichoderma reesei QM6a provides the basis for inbreeding in this industrial cellulase producing fungus
Source: Biotechnol Biofuels. 2015 Sep 24;8:155. doi: 10.1186/s13068-015-0311-2 (PMC4581161; doi:10.1186/s13068-015-0311-2)
Supplement: Additional file 5: — Table S3. Fifty best BLAST hits (NCBI) for Trire2:67350 using the protein sequence of RL1/A8-02 derived from cDNA sequencing. [file 13068_2015_311_MOESM5_ESM.docx]

**Supplementary Table S3:** Fifty best BLAST hits (NCBI) for Trire2:67350 using the protein sequence of RL1/A8-02 derived from cDNA sequencing.

| **Hit** | | **identity** | **positives** | **e-value** | **bit score** |
| --- | --- | --- | --- | --- | --- |
| **encoded protein** | **accession number** |  |  |  |  |
| hypothetical protein M419DRAFT_87315 [Trichoderma reesei RUT C-30] | ETR98956.1 | 92% | 1501 | 0.0 | 2996 |
| predicted protein [Trichoderma reesei QM6a] | XP_006968234.1 | 92% | 1500 | 0.0 | 2995 |
| hypothetical protein TRIVIDRAFT_50286 [Trichoderma virens Gv29-8] | EHK26592.1 | 83% | 1417 | 0.0 | 2566 |
| hypothetical protein TRIATDRAFT_221851 [Trichoderma atroviride IMI 206040] | EHK45402.1 | 77% | 1097 | 0.0 | 1934 |
| hypothetical protein S40288_00598 [Stachybotrys chartarum IBT 40288] | KFA79026.1 | 64% | 1274 | 0.0 | 1919 |
| hypothetical protein S7711_06878 [Stachybotrys chartarum IBT 7711] | KEY66927.1 | 64% | 1274 | 0.0 | 1919 |
| hypothetical protein S40285_02965 [Stachybotrys chlorohalonata IBT 40285] | KFA67096.1 | 64% | 1267 | 0.0 | 1914 |
| WD40 domain protein [Metarhizium robertsii] | EXU99511.1 | 70% | 1102 | 0.0 | 1833 |
| hypothetical protein FVEG_11510 [Fusarium verticillioides 7600] | EWG52933.1 | 62% | 1199 | 0.0 | 1807 |
| hypothetical protein FPSE_07566 [Fusarium pseudograminearum CS3096] | EKJ72253.1 | 63% | 1212 | 0.0 | 1802 |
| hypothetical protein ACRE_069200 [Acremonium chrysogenum ATCC 11550] | KFH42369.1 | 60% | 1225 | 0.0 | 1801 |
| hypothetical protein FOTG_16711 [Fusarium oxysporum f. sp. vasinfectum 25433] | EXM14912.1 | 60% | 1168 | 0.0 | 1751 |
| hypothetical protein FOMG_16862 [Fusarium oxysporum f. sp. melonis 26406] | EXK26537.1 | 59% | 1168 | 0.0 | 1743 |
| hypothetical protein FOIG_07738 [Fusarium oxysporum f. sp. cubense tropical race 4 54006] | EXM00846.1 | 67% | 1053 | 0.0 | 1727 |
| hypothetical protein FOMG_08546 [Fusarium oxysporum f. sp. melonis 26406] | EXK38039.1 | 67% | 1052 | 0.0 | 1726 |
| hypothetical protein FOTG_06907 [Fusarium oxysporum f. sp. vasinfectum 25433] | EXM26639.1 | 67% | 1052 | 0.0 | 1725 |
| hypothetical protein FOCG_14672 [Fusarium oxysporum f. sp. radicis-lycopersici 26381] | EXL43196.1 | 67% | 1052 | 0.0 | 1724 |
| hypothetical protein FOZG_14953 [Fusarium oxysporum Fo47] | EWZ31927.1 | 67% | 1051 | 0.0 | 1724 |
| uncharacterized protein FFUJ_08367 [Fusarium fujikuroi IMI 58289] | CCT71549.1 | 67% | 1050 | 0.0 | 1724 |
| hypothetical protein FOWG_04778 [Fusarium oxysporum f. sp. lycopersici MN25] | EWZ94516.1 | 67% | 1051 | 0.0 | 1722 |
| hypothetical protein FOC4_g10004597 [Fusarium oxysporum f. sp. cubense race 4] | EMT73231.1 | 60% | 1167 | 0.0 | 1716 |
| hypothetical protein FOC1_g10009666 [Fusarium oxysporum f. sp. cubense race 1] | ENH72930.1 | 60% | 1167 | 0.0 | 1715 |
| IDC1 protein [Villosiclava virens] | KDB13870.1 | 60% | 1131 | 0.0 | 1713 |
| hypothetical protein FGSG_10835 [Fusarium graminearum PH-1] | ESU17936.1 | 67% | 1057 | 0.0 | 1706 |
| hypothetical protein FOQG_15732 [Fusarium oxysporum f. sp. raphani 54005] | EXK79707.1 | 60% | 1126 | 0.0 | 1687 |
| hypothetical protein FOC1_g10000376 [Fusarium oxysporum f. sp. cubense race 1] | ENH65203.1 | 60% | 1131 | 0.0 | 1656 |
| hypothetical protein FOTG_16673 [Fusarium oxysporum f. sp. vasinfectum 25433] | EXM14971.1 | 59% | 1123 | 0.0 | 1644 |
| hypothetical protein GLRG_05175 [Colletotrichum graminicola M1.001] | EFQ30031.1 | 58% | 1172 | 0.0 | 1644 |
| WD domain, G-beta repeat protein [Cordyceps militaris CM01] | XP_006667621.1 | 58% | 1156 | 0.0 | 1642 |
| hypothetical protein CSUB01_00857 [Colletotrichum sublineola] | KDN62152.1 | 59% | 1180 | 0.0 | 1640 |
| hypothetical protein CFIO01_05485 [Colletotrichum fioriniae PJ7] | XP_007589725.1 | 59% | 1181 | 0.0 | 1630 |
| hypothetical protein MANI_009668 [Metarhizium anisopliae] | KFG81794.1 | 64% | 1022 | 0.0 | 1618 |
| IDC1 protein [Beauveria bassiana ARSEF 2860] | XP_008602603.1 | 64% | 1030 | 0.0 | 1593 |
| hypothetical protein NECHADRAFT_93326 [Nectria haematococca mpVI 77-13-4] | XP_003048179.1 | 58% | 1139 | 0.0 | 1590 |
| hypothetical protein FOYG_17378 [Fusarium oxysporum FOSC 3-a] | EWY79473.1 | 64% | 994 | 0.0 | 1586 |
| WD g-beta repeat protein [Colletotrichum orbiculare MAFF 240422] | ENH87883.1 | 56% | 1152 | 0.0 | 1582 |
| hypothetical protein MAA_02610 [Metarhizium anisopliae ARSEF 23] | XP_007818799.1 | 64% | 1013 | 0.0 | 1578 |
| hypothetical protein MAC_03576 [Metarhizium acridum CQMa 102] | XP_007809916.1 | 63% | 999 | 0.0 | 1539 |
| hypothetical protein FG10835.1 [Fusarium graminearum PH-1] | XP_391011.1 | 63% | 992 | 0.0 | 1531 |
| hypothetical protein FOWG_17010 [Fusarium oxysporum f. sp. lycopersici MN25] | EWZ78760.1 | 60% | 1010 | 0.0 | 1528 |
| hypothetical protein MGG_06673 [Magnaporthe oryzae 70-15] | XP_003709379.1 | 54% | 1099 | 0.0 | 1484 |
| hypothetical protein FOWG_17591 [Fusarium oxysporum f. sp. lycopersici MN25] | EWZ78091.1 | 56% | 1059 | 0.0 | 1481 |
| idc1 protein [Colletotrichum gloeosporioides Nara gc5] | XP_007274016.1 | 58% | 1014 | 0.0 | 1467 |
| IDC1 protein [Verticillium dahliae VdLs.17] | EGY18713.1 | 54% | 1111 | 0.0 | 1459 |
| hypothetical protein GGTG_13866 [Gaeumannomyces graminis var. tritici R3-111a-1] | EJT68561.1 | 53% | 1073 | 0.0 | 1449 |
| IDC1 protein [Neurospora crassa OR74A] | ESA43608.1 | 52% | 1053 | 0.0 | 1414 |
| hypothetical protein PFICI_08975 [Pestalotiopsis fici W106-1] | XP_007835747.1 | 58% | 977 | 0.0 | 1412 |
| uncharacterized protein CPUR_00378 [Claviceps purpurea 20.1] | CCE26909.1 | 59% | 936 | 0.0 | 1403 |
| hypothetical protein SMAC_02471 [Sordaria macrospora k-hell] | XP_003350801.1 | 54% | 979 | 0.0 | 1380 |
| IDC1 protein [Pseudallescheria apiosperma] | KEZ43835.1 | 54% | 1064 | 0.0 | 1379 |
