# Supplementary material for: Restoration of female fertility in Trichoderma reesei QM6a provides the basis for inbreeding in this industrial cellulase producing fungus
Source: Biotechnol Biofuels. 2015 Sep 24;8:155. doi: 10.1186/s13068-015-0311-2 (PMC4581161; doi:10.1186/s13068-015-0311-2)
Supplement: Additional file 7: — Figure S3. Nucleotide sequence of the ham5 locus from T. reesei QM6a and T. reesei RL1/A8-02 including the mutation being critical for female fertility. The sequence differences are marked in gray, introns are underlined. The critical mutation (G- > T) in the second intron of strain QM6a is indicated by a red box and the resulting stop codon is labeled in yellow. [file 13068_2015_311_MOESM7_ESM.docx]

QM6a ATGCCGTCCGCCGCGGATCTCAAATACTTCATCCCGTCCGCTGCCACGGCGTCCATCTTC

RL1/A8-02 ATGCCGTCCGCCGCGGACCTCAAATACTTCATCCCGTCTGCTGCCACGGCGTCCATCTTC

QM6a CTCTATGCCCAGGGACCAACGATAGTCTGTTGCCATCACGACACGCTCACCATCGACCGG

RL1/A8-02 CTCTATGCGCAGGGACCAACGATAGTCTGTTGCCATCACGACACGCTCACCATCGACCGG

QM6a AGGTTCTCACGCCATGTCGACGATGTCCAGCTGCTTGCCGTGGATAACCAGAGCGACATG

RL1/A8-02 AGGTTCTCACGCCATGTCGACGATGTCCAGCTGCTTGCCGTGGATAACCAGAGCGACATG

QM6a GGCGCTGGCCGGTTCGTCGTGAGCTATGACGCTGGCCAGACTGCCATTGTGTGGGATCTA

RL1/A8-02 GGCGCTGGCCGGTTCGTCGTGAGCTATGACGCTGGCCAGACTGCCATTGTGTGGGATCTA

QM6a ATGACGGGCGATGAGATTTCGCGTTTCGTTTCGTACGAGACGCTCACAGTCGCCGCTTGG

RL1/A8-02 ATGACGGGCGATGAGATTTCGCGTTTCGTTTCGTACGAGACGCTCACAGTCGCCGCTTGG

QM6a ATGCGGAATGGGAACGTTGCTTTCGGTGAGCGGAGCCAGAGAT--GCCCCCCCTCCTCTC

RL1/A8-02 ATGCGGAATGGAAACGTTGCTTTCGGTGAGCGGAACCAGAGATCGCCCTCCCCCTCTCCC

QM6a CTTAAGCTGGTGACATGAGGTGGCTAACGGATTGGGCTTCCAGGCAACACACAGGGAAAC

RL1/A8-02 CTTAAGCTGGTGACATGAGGTGGCTAACGGATTGGGCTTCCAGGCAACACACAGGGAAAC

QM6a ATCATCATGTTTGAACCTACTACATCCGAGCATATCTCGGCTCGGACGCTAGACCAGATT

RL1/A8-02 ATCATCATGTTTGAACCTACTACATCCGAGCATATCTCGGCTCGGACGCTAGACCAGATT

QM6a GCAGTGACAGCATTAGCCCCTTCGTCTGACTGCCGGACCTTTGCCATTGGTTAAGTTGGT

RL1/A8-02 GCAGTGACAGCATTAGCCCCTTCGTCTGACTGCCGGACCTTTGCGATCGGGTAAGTTGGT

QM6a GTTCGACCATGAATCGAGAAGTCTGTCGCGAATGTGCCACGGCGTACTGATTCCATCCGA

RL1/A8-02 GTTCGACCATGAATCGAGAAGTCTGTCGCGAATGTGCCACGGCGTACTGATTCCATCCGA

QM6a TGTAGCTACCAGAACGGATCTCTCTTGGTCGCAACTCTGCAGCCTCGGTTCACCATCTTA

RL1/A8-02 TGTAGCTACCAGAACGGATCTCTCTTGGTCGCAACTCTGCAGCCTCGGTTCACCATCTTA

QM6a CACAACCTGACGACCTCGAGAGGGCCATCGCCCATTGTCACCCTCGCCTGGCACGCGTCC

RL1/A8-02 CACAACCTGACGACCTCGAGAGGGCCATCGCCCATTGTCACCCTCGCCTGGCACGCGTCC

QM6a TCCTCGAGGCAGAAATCAGACATGCTGGCCGTGCAGACGCACGATGGCGACCTGCGGGTC

RL1/A8-02 TCCTCGAGGCAGAAATCAGACATGCTGGCCGTACAGACGCACGATGGCGACCTGCGGGTC

QM6a TGGAGTGTGGCCAAGTCGTACAGCGCCGAAGATCCCGCCAAGGTCGTCAGGGTGCTCCGC

RL1/A8-02 TGGAGTGTGGCCAAGTCGTACAGCGCCGAAGATCCCGCCAAGGTCGTCAGGGTGCTCCGC

QM6a AGGAACGAGAACTTCCTGGCTGGGCCCAATTGGATGGGGTGGTCCAAGAACGGCCGCATC

RL1/A8-02 AGGAACGAGAACTTCCTGGCTGGGCCCAATTGGATGGGGTGGTCCAAGAACGGCCGCATC

QM6a ATCCAATACTCGGACTCGTAAGTCCCCTATCAAGTGGACACAGCCTAAGCGCGCGGCCTC

RL1/A8-02 ATCCAATACTCGGACTCGTAAGTCCCCTATCAACTGGACACAGCCTAAGCGCGCGGCCTC

QM6a CCGCTTCTGCTGGTTTGGTTTTTTGTTGTGCTGACCTCGTTGTGCAGGGAAACGTTTTCG

RL1/A8-02 CCGCTTCTGCTGGTTTGGTTTTTTGTTGTGCTGACCTCGTTGTGCAGGGAAACGTTTTCG

QM6a TGGGACGTGAGAACCAAACATGTCACAAAGGACTCCATCCCGACCCTTGAGCATGTTAAA

RL1/A8-02 TGGGACGTGAGAACCAAACATGTCACAAAGGACTCCATCCCGACCCTTGAGCATGTTAAA

QM6a GGCCTGGCCGTCTATGGCCCAGGAGCCAGTCTCTTCACCCTTGGACCGAACAACACCGTG

RL1/A8-02 GGCCTGGCCGTCTATGGCCCAGGAGCCAGTCTCTTCACCCTTGGACCGAACAACACCGTG

QM6a CAGCAGTTTGACCTCAACTCCCCAGCCATCATGGTGGCAAACGTGCAGCACCCCGCGGGC

RL1/A8-02 CAGCAGTTTGACCTCAACTCCCCAGCCATCATGGTGGCAAACGTGCAGCACCCCGCGGGC

QM6a GTCCTTCCGCCATCTCCACCCACCTCCGAGGAGACAGGGGGCAGGTCGGTGCACTCTGCC

RL1/A8-02 GTCCTTCCGCCATCTCCACCCACCTCCGAGGAGACAGGGGGCAGGTCGGTGCACTCTGCC

QM6a ACGACCATTCATACCTCGGAATCCGAGTCGAGCTCTGTCCCCCTCGAGATGGGCATCTCT

RL1/A8-02 ACGACCATTCATACCTCGGAATCCGAGTCGAGCTCTGTCCCCCTCGAGATGGGCATCTCT

QM6a GAAAGCGATGACGACCACCTATCTCCTTTCCAGCGGTTAGCAAAGCGCAATGCCCCCGAA

RL1/A8-02 GAAAGCGATGACGACCACCTATCCCCTTTCCAGCGGCTAGCAAAGCGCAATGCCCCCGAA

QM6a GCCAGGCACGAGGTGTACGATACAGGCAGCGCGGCCTCTAGCCAGAGCGGCGTGTCATCC

RL1/A8-02 GCCAGGAACGAGGTGTACGATACAGGCAGCGCGGCCTCTAGCCAGAGCGGCGTGTCATCC

QM6a TTGTCAAAGTCTTCGGCCAGCTCTCGTACGCCTGGCAGGCAGGCCAGCTCCCTCAGGTCG

RL1/A8-02 TTGTCAAAGTCTTCGGCCAGCTCTCGTACGCCTGGCAGGCAGGCCAGCTCGCTCAGGTCG

QM6a CGGGGAATGACGGAGGGGACATACATCTCCGCTGGTTCATCCATGAAAACTTCGACAGTC

RL1/A8-02 CGGGGAATGACGGAGGGGACATACATCTCCGCTGGTTCATCCATGAAAACTTCGACAGTT

QM6a GGCCAGCGCGAGGCGGACAACTACTCCATGGGATACACACTCCCTAGCACCAGCGGCCCA

RL1/A8-02 GGCCAGCGCGAGGCGGACAACTACTCCATGGGATACACACTCCCTAGCACCAGCGGTCCA

QM6a TCATTGGCGTCGTCCCGGTCCAGGCATCGGCCCTCTCGCCTCAGACACGAAGTACCGAGG

RL1/A8-02 TCATTGGCGTCGTCCCGGTCCAGGCATCGGCCCTCTCGTCTCAGACACGAAGTACCGAGG

QM6a AGTCCTGACGAGGCCAACGTGCAGGATCTGTTCAAATACACTCGCTCGCGGCTCAGCGAC

RL1/A8-02 AGTCCTGACGAGGCCAACGTGCAGGACCTGTTCAAATACACTCGCTCGCGGCTTAGCGAC

QM6a CTCCCGTACAAGCACCCGATGCCAACGCAACGATCTCATCCTACCAATGACGATTTGCGC

RL1/A8-02 CTCCCGTACAAGCACCCGATGCCAACGCAACGATCTCATCCTACCAATGACGATTTGCGC

QM6a CGACAGATGCTCAGCACTATCTTTGGCTGGAACAAGGAAGTAGAAGACCTCATTCGAGAC

RL1/A8-02 CGACAGATGCTCAGCACTATCTTTGGCTGGAACAAGGAAGTAGAAGACCTCATTCGAGAC

QM6a GAGATGAGTCGCTATCCCGCAGGGTCGGCGAACCGAATCTTGCTAGCCAAGTGGCTGGGC

RL1/A8-02 GAGATGAGTCGCTATCCCGCAGGGTCGGCGAACCGAATCTTGCTAGCCAAGTGGCTGGGC

QM6a GACATTGACGCAGACATCATGGCCGCGGGTTCCCAGAACATGACTTCCCAAGACTGGATG

RL1/A8-02 GACATTGACGCAGACATCATGGCCGCGGGTTCCCAGAACATGACTTCCCAAGACTGGATG

QM6a CTGCTGGCACTGAGCGGCATTGGCGGCCAGGCATCCCAGCACAAGCTCGGCCGGGTCTAT

RL1/A8-02 CTGCTGGCACTGAGCGGCATTGGCGGCCAGGCATCCCAGCACAAGCTCGGCCGAGTCTAT

QM6a GTCCAGCGCCTGCTAGAGAATGGCGATGTCCATGTTGCGGTGACGATTATGCTTGGGATG

RL1/A8-02 GTCCAACGCTTGCTAGAGAATGGCGATGTCCATGTTGCGGTGACGATTATGCTTGGGATG

QM6a GGCGACTATAACGATGCCATTGAGGTCTACATCTCGCACAAGCGGTATATGGAGGCCCTC

RL1/A8-02 GGCGACTATAACGATGCCATTGAGGTCTACATCTCGCACAAGCGGTATATGGAGGCCCTC

QM6a ATTCTCACCTGCGTGGCTTTTCCCAGCGTCTGGGAGCGTCAAGCTGCCATTGTGCGCAAA

RL1/A8-02 ATTCTCACCTGCGTGGCTTTTCCCAGCGTCTGGGAGCGTCAAGCTGCCATTGTGCGCAAA

QM6a TGGGGCGAGTGGGCGGTCAAGCATGGCCAGCAGCAACTGGCAATTCGTTGCTTTGCCTGT

RL1/A8-02 TGGGGTGAGTGGGCGGTCAAGCATGGCCAGCAGCAACTGGCAATTCGTTGCTTTGCCTGT

QM6a ACCGACCAGGAGTCCTCGGAGCCCTGGACCTCTCCATCGGCTGCCCAGCTCAACTTCCAA

RL1/A8-02 ACCGACCAGGAGTCCTCGGAGCCTTGGACCTCTCCATCGGCTGCCCAGCTAAACTTCCAA

QM6a AACATCACCCCGAGCATCCCCGAGGTGCTGAGCCCTCCGCTGTCGCCCCCGGGCATCCAG

RL1/A8-02 AACATCACCCCGAGCATCCCCGAGGTGCTGAGCCCTCCGCTGTCGCCCCCGGGCATTCAG

QM6a AGGGGCCCCCAGCGCAGCGTCGCCAAGGCATCGGCACTGAAGCTGATTACGTCCTTTGGA

RL1/A8-02 AGAGGCCCCCAGCGCAGCGTCGCCAAGGCATCGGCACTGAAGCTGATTACGTCCTTTGGA

QM6a GATCCCACCCAAAAGGCCAAGTTCTACTCGCAGGCTGACGGCGGCCAGACACCGATTGCA

RL1/A8-02 GATCCCACCCAAAAGGCCAAGTTCTACTCGCAGGCTGACGGCGGCCAGACACCAATTGCA

QM6a GCTGGAGTGACGCCCATTGCCGAGTCGGCCATCTCTCCGGGAGGCGCCTATGATCCCGCG

RL1/A8-02 GCTGGAGTGACGCCCATTGCCGAGTCGGCCATCTCTCCGGGAGGCGCCTATGATCCCGCA

QM6a ACTGCGTTCCTCCGGCCGTCGGGCAACAGCAGGTTCAACACGCCAACGTCAGCCCGCCCT

RL1/A8-02 ACTGCGTTCCTCCGGCCGTCGGGCAATAGCAGGTTCAACACGCCAACATCAGCCCGCCCT

QM6a ATCGGTCAGGGCTTCAGCCGCGGACGGTTGCCCTCCATCGGGGAAGCCAACAAGCCCCTG

RL1/A8-02 ATCGGTCAGGGCTTCAGCCGCGGACGGCTGCCCTCCATCGGAGAAGCCAACAAGCCCCTG

QM6a GATAACATTGCAAGCATCGTGGACGCACCCCAGAAACGGCCGAGCCATTCTCGCAAGTCG

RL1/A8-02 GATAACATTGCAAGCATTGTGGACGCACCCCAGAAACGGCCGAGCCATTCTCGCAAGTCG

QM6a TCCGCCCCTCAAGATAACATGGCGACCGGGCTCGCCATGCAGCGCGCTGCTACGGCCAGC

RL1/A8-02 TCCGCCCCTCAAGATAACATGGCAACCGGGCTCGCCATGCAGCGCGCTGCTACGGCCAGC

QM6a CCAATGATGATGAGGGACCAGTACCAGCGCGCCGTGCAAGGGTACGGAGGAGAGCGACCG

RL1/A8-02 CCAATGATGATGAGGGACCAGTACCAGCGCGCCGTGCAAGGGTACGGAGGAGAGCGACCG

QM6a CCGTCTCCCGACCATAACATCATGAGCAGGCTGCAAGAAGTCCATTCGGCACAGCGAAAC

RL1/A8-02 CCGTCTCCCGACCATAACATCATGAGCAGACTGCAAGAGGTCCATTCGGCACAGCGAAAC

QM6a GGCTCCCGTGACCGGATTCCTGCTCACCTCAGCCTGCAGCTGCAGACGATGCAGCCGCCA

RL1/A8-02 GGCTCCCGTGACCGGATTCCTGCTCACCTTAGCCTGCAGCTGCAGACGATGCAGCCGCCA

QM6a TCAATGGATGCAATGTCTCCTGAGCAATCTGGCGCCTCATCCGCTCGCTTCCACTGGCCG

RL1/A8-02 TCGATGGAGGCAATGTCTCCTGAGCAATCCGGCGCCTCATCCGCTCGCTTCCACTGGCCG

QM6a TCTCGTCGCCGCGGTACTGGCCCTAGTTCCGCATCAGTGGCAGGTTCCATGACATCCACC

RL1/A8-02 TCTCGTCGCCGCGGTACTGGTCCTAGTTCCGCATCTGTGGCCGGTTCCATGACATCCACC

QM6a TCCAGCGCTGGTCGGAGTCACAAATCGAACGCTCGACAAAGGGATGATTACATCCATAGC

RL1/A8-02 TCCAGCGCTGGCCGGAGTCACAAATCGAACGCTCGACAAAGGGATGATTACATCCATAGC

QM6a CTGGAAGCGGCTCAGCACTATTCCAGGAGAGCTGGAAGTCGGACCGGAAGCAAGGAACGG

RL1/A8-02 CTGGAAGCGGCTCAGCACTATTCCAGGAGAGCTGGAAGCCGGACCGGAAGCAAGGAACGG

QM6a ACACGAGACGCGTCCACCGGCCGCCACGCCAGCCGAGAGCGACGAACCAAGTCGCGCGAC

RL1/A8-02 ACACGAGACGCGTCCACCGGCCGCCACGCCAGCCGAGAGCGACGAACCAAGTCGCGCGAC

QM6a CCTTCGGAAGACCGCGGTAGGGCTTCAGCCAGATCCTGGACAAGGCCAAAGCGATCGCCC

RL1/A8-02 CCTTCGGAAGAGCGCGGCAGGGCTTCAGCCAGATCCTGGACAAGGCCAAAGCGATCACCC

QM6a ACATCCCCAGTTCCAATGTCCCCCGAAGACTTGGCCATGCTGAGCAACCGGACATTCGAC

RL1/A8-02 ACATCCCCAGTTCCAATGTCCCCCGAAGACTTGGCCATGCTGAGCAACCGGACATTCGAC

QM6a AACTCCGTGGAACCCCTCACGATTCGAAAGGCGAGCGTGGCGAAAGGCAAGACCAGCGGG

RL1/A8-02 AACTCCGTGGAACCCCTCACGATTCGAAAGGCGAGCGTGGCGAAAGGCAAGGCCAGCGGG

QM6a CGGACATCCAGCCGCGGAAGGGGTGGATCAGTACCGCGGTCACCGCCGTCCCCGGTGCCG

RL1/A8-02 CGGACATCCAGCCGCGGAAGGGGTGGATCAGTACCGCGGTCACCGCCGTCCCCGGTGCCG

QM6a CTGTCGGCAACAGCCCTGCATTATCAGGGATCGGAGGACGAGGAGGACTTTAGAGCAGCC

RL1/A8-02 CTGTCGGCAACAGCCCTGCATTATCAGGGATCGGAGGACGAGGAGGACTTTAGAGCAGCC

QM6a ATGCGGGCGCAGGAAGAGTTCAGGGCCAAGCACAGCCGCAGCGTCGGCCACAACGTCAAT

RL1/A8-02 ATGCGGGCGCAGGAAGAGTTCAGGGCCAAGCACAGCCGCAGCGTCGGCCACAACGTCAAT

QM6a TCTCCCGCCGTAAGCCGGCGTGAGCATTCGGAAAGCCGACGAAAGGAAGCGACTGAGACT

RL1/A8-02 TCTCCCGCCGTAAGCCGGCGTGAGCATTCGGAAAGCCGACGAAAGGAAGCGACTGAGACT

QM6a CGAGAGGCTGCACCCGTGGTCTTGAGCCAGACAGTATATGGACGAGCCGCTTCCACCGAG

RL1/A8-02 CGAGAGGCTGCACCCGTGGTCTTGAGCCAGACAGTATATGGACGAGCCGCTTCCACCGAG

QM6a CACGCCGGCGATCTGAAGAAGATGAAGGACGAGAGACAGCGGAAGAAGGAACAGGCTGCG

RL1/A8-02 CACGCCGGCGATCTGAAGAAGATGAAGGACGAGAGACAGCGGAAGAAGGAACAGGCTGCG

QM6a CGAGAGCTAGAAGAGCGTAGGAAGTCTCTGGCCAAGCGACAGCTCGGTCCCCGAATTCCC

RL1/A8-02 CGAGAGCTAGAAGAGCGTAGGAAGTCTCTGGCCAAGCGACAGCTCGGTCCCCAGATTCCC

QM6a CATCCAAGCCAGATCTCTCCCGGGAGACCGCCGGTTCTGGTAGAGGCCGACGACGAAAAG

RL1/A8-02 CATCCAAGCCAGATCTCTCCCGGGAGACCGCCGGTTCTGGTAGAGGCCGACGATGAAAAG

QM6a CTGCCGGATGATCTGCCGCCGCGCTCTGCAACGGAGCCTCCGAGAGCGGCGGAGCCGCCG

RL1/A8-02 CTGCCGGATGATCTGCCGCCGCGCTCTGCAACAGAACCCCCGAGGACGGCGGAGCCGCCG

QM6a AGGAGCATGTATGCTCAGAACAGGCCGCAGATTGGCCTGCCTGCCACTCCCAAGGCGATG

RL1/A8-02 AGGAGCATGTATGCTCAAAACAGGCCGCAGATTGGCCTGCCTGCCACTCCCAAGGCGATG

QM6a AGGCTCATTATCCGGTCTGACGAGAATCAGTACGAGGATCTCCCTGCTCCGCCGGTGCCG

RL1/A8-02 AGGCTCATTATCCGGTCTGACGAGAATCAGTACGAGGACCTCCCTGCTCCGCCGGTGCCG

QM6a GCGACGTTTTCTCAAAAGTATTCGCCGCAGAACTCACCCCAGTACTCTCCAAAATACACG

RL1/A8-02 GCGACGTTTTCTCAAAAGTATTCGCCGCAGAACTCACCCCAGTACTCTCCGAAATACACG

QM6a TTGGGCAGTAGTGTTTATGGAGAGGAGCAGAAGCAGCAGCAGCAGGGACAGCAGCAGCAG

RL1/A8-02 TTGGGCAGTAGTGTTTATGGAGAGGAGCAGAAGCAGCAGCAGCAGCAGCAGCAACAGCAG

QM6a CAACAACGACAACAACAACAGCAGCAGCAACAACAACAGCAGCAGCAACAACAACAGCAG

RL1/A8-02 GGACA---------------GCAGCAGCAGCAACAACGACAGCAACAACAACAGCAGCAG

QM6a CAGCAACAGCAACAGCAGCAGCAGCAGCAACAACAACAACTCTTTCAGCAGAAAGAGGAG

RL1/A8-02 CAGCAACAGCAACAGCAGCAGCAGCA---ACAACAACAACTCTTTCATCAGAAAGAGGAG

QM6a GAGCCGCCGTTGACACTGCTGCCATCGACTGTCTATCAGCTGCCGTCCACTGTCTACCAA

RL1/A8-02 GAGCCGCCGTTGACACTGCTGCCATCGACTGTCTATCAGCTGCCGTCCACTGTCTACCAA

QM6a CCGCCGTCTCGCCCCATGATTCCGCGAAGCATGTCGGCACCGATCCCTGATGAGCCACCT

RL1/A8-02 CCGCCGTCTCGCCCCATGATTCCGCGAAGCATGTCGGCACCGATCCCTGATGAGCCACCT

QM6a CACGCAATTCGATACGGGAGAAAGTCGAGTGCTAACGAGGGCAGAGGCCTCGACGATATT

RL1/A8-02 CACGCAATTCGATACGGGAGAAAGTCGAGTGCTAACGAGGGCAGAGGCCTCGACGATATT

QM6a GTAGAGACGGAGTGGCGTCGACAAGTGAACAACCTGGTGCCGCCTCCACCACCACCTCCC

RL1/A8-02 GTAGAGACGGAGTGGCGTCGACAAGTGAACAACCTGGTGCCGCCTCCACCACCACCTCCC

QM6a CCTTCGGCTCCCCTGACTTTCCTCAAGGAGCTGCAGCATCTAGCCGTGCCGCCCCCTCCT

RL1/A8-02 CCTTCGGCGCCCCTGACTTTCCTCAAGGAGCTGCAGCATCTAGCCGTGCCGCCCCCTCCT

QM6a CCGCCAGCACCGCTGCCACACGTGCGACGCCAGCCGCCCGTGGCTGGAACACTGGCCTCG

RL1/A8-02 CCGCCAGCACCGCTGCCACACGTGCGACGCCAGCCGCCCGTGGCTGGAACACTGGCCTCG

QM6a GGCATGATTGAGATTGTCATGGACGACGACGAGGCAGAAGAGCCCATGTCGGCTGCTCCC

RL1/A8-02 GGCATGATCGAAATTGTCATGGACGATGACGAGGCAGAAGAGCCCATGTCGGCTGCTCCC

QM6a AACGACGGCAAGGTGCCGGTGCTCGCCCAGCCCGAACCCCCTGTCAAGGGCCACAACCGC

RL1/A8-02 AACGACGGCATGGTGCCGGTGCTCGCCCAGCCCGAACCCCCTGTCAAGGGCCACAACCGC

QM6a GGTCGCAGCCTCGGGGAGGGCAGCCTTTCCGGGCGTAGGACCAAGGCGACGGAGCGCCTC

RL1/A8-02 GGTCGCAGCCTCGGGGAGGGCAGCCTTTCCGGGCGTAGGACCAAGGCGACGGAGCGCCTC

QM6a CGCTCGGGCAGCCGCAGCCGCAAAGGATCCATCGGCGTCATGTCGCCCCCTCTCGAGATG

RL1/A8-02 CGCTCGGGCAGCCGCAGCCGCAAAGGATCCATCGGCGTCATGTCGCCCCCTCTCGAGATG

QM6a TACGGCGGTGAGGGCAATGCCAACTCGCTGAACCAGCTCAGGTCGCCCGTGGCGGGCAAT

RL1/A8-02 TACGGCGGTGAGGGCAATGCCAACTCGCTGAACCAGCTCAGGTCGCCCGTGGTGGGCAAT

QM6a CCTCCGCCTGTGCTGTATGACCGGGACGCCATTCGATCGCCCATTGAGGGGCATGGCCGC

RL1/A8-02 CCTCCGCCTGTGCTGTATGACCGGGACGCCATTCGATCGCCCATTGAGGGGCATGGCCGC

QM6a AAGATGTCGATGGGGCTGCACGAGAACGGCATGTTTTAG

RL1/A8-02 AAGATGTCGATGGGGCTGCACGAGAACGGCATGTTTTAG
