# Supplementary material for: Restoration of female fertility in Trichoderma reesei QM6a provides the basis for inbreeding in this industrial cellulase producing fungus
Source: Biotechnol Biofuels. 2015 Sep 24;8:155. doi: 10.1186/s13068-015-0311-2 (PMC4581161; doi:10.1186/s13068-015-0311-2)
Supplement: Additional file 8: — Table S4. Sequences of the primers used for mating-type determination, the vector construction for the direct MAT locus replacement in T. reesei QM6a MAT1-2, the construction of the deletion cassettes for Trire2:59270 and Trire2:67350 and the plasmid construction for gene complementation in T. reesei QM6a MAT1-1. [file 13068_2015_311_MOESM8_ESM.docx]

**Supplementary Table S4:** Sequences of the primers used for mating-type determination, the vector construction for the direct MAT locus replacement in *T. reesei* QM6a *MAT1-2*, the construction of the deletion cassettes for Trire2:59270 and Trire2:67350 and the plasmid construction for gene complementation in *T. reesei* QM6a *MAT1-1*.

| **Primer** | **Sequence** |
| --- | --- |
| MAT1-1-1 fw (for diagnostic PCR) | CTTCCTACACGGATGCCAGA |
| MAT1-1-1 rv (for diagnostic PCR) | CTCGAGAGGGATATACACCAG |
| MAT1-1 fw | GTGCTGGAATTCAGGCCTGGCTTGATGCTGCTAACCTTC |
| MAT1-1 rv | TCTGCAGAATTCAGGCCTACTCCGCAAGATCAAATCCG |
| hph_AvrII_fw | GTCCACAGAAGAGCCTAGGACCTCTTCGGCGATACATACTC |
| hph_AvrII_rv | GGCTTTCACGGACCCTAGGTTGGAATCGACCTTGCATG |
| 1-2_replace_ cassette_fw | TGGAACGACTTTGTACGCAC |
| 1-2_replace_ cassette_rv | GGCACAAGAGGACAGACGAC |
|  |  |
| 59270_5F | GTAACGCCAGGGTTTTCCCAGTCACGACGGAGGATCAACAGTCTACAGC |
| 59270_5R | CATATTGATGTAAGGTAGCTCTCGGATCCACCACCCCACTAAGATAAGG |
| 59270_3F | TATTCCATCTAAGCCATAGTACCCTCGAGGGTAGGTAGGTAGCTCATGC |
| 59270_3R | GCGGATAACAATTTCACACAGGAAACAGCGAGTGTCATGTGAGACAACC |
| 67350_5F | GTAACGCCAGGGTTTTCCCAGTCACGACGCCTAGCCTGCTCTTATTACC |
| 67350_5R | CATATTGATGTAAGGTAGCTCTCGGATCCCAAGCTCGTGAGACAGTACC |
| 67350_3F | TATTCCATCTAAGCCATAGTACCCTCGAGCCTTTGTCTTCTCTTCGTTCG |
| 67350_3R | GCGGATAACAATTTCACACAGGAAACAGCGAGATGACACTTCAGGGAGG |
| hph_fw | GGATCCGAGAGCTACCTTAC |
| hph_rv | CTCGAGGGTACTATGGCTTA |
|  |  |
| 59270_complement_fw | TAAAACGACGGCCAGTGAATTCCGCCAGGTTGATCTTGTTCTAC |
| 59270_complement_rv | AGGAAATCAAAGCCGTCTAGATCGAAGTGTAGGCTGGAATGAG |
| 67350_complement_fw | TAAAACGACGGCCAGTGAATTCAATAAAGACGGCCTGGAAAC |
| 67350_complement_ rv | AGGAAATCAAAGCCGTCTAGATTCCGCATACCACCTACTTG |
| 3422_complement_fw | TAAAACGACGGCCAGTGAATTCGTGTATGAAGCACTCGCATCTC |
| 3422_complement_ rv | AGGAAATCAAAGCCGTCTAGAAATGCGGGTTTAAAGACGAC |
| 47930_complement_fw | TAAAACGACGGCCAGTGAATTCGCTGGCACTGGATGGTATAAAC |
| 47930_complement_ rv | AGGAAATCAAAGCCGTCTAGAGCTTCCTTCCGACCATCTTTAC |
| 81593_complement_fw | TAAAACGACGGCCAGTGAATTCCGATGCTGCATCTGTAAATGTC |
| 81593_complement_ rv | AGGAAATCAAAGCCGTCTAGATCGGAGCTGCCAATGTTTC |
| in geneticin rv | ATCCCGAAAGCATCACCG |
